# Solar energy conversion by photosystem II: principles and structures

**DOI:** 10.1007/s11120-022-00991-y

**Published:** 2023-02-24

**Authors:** Dmitry Shevela, Jan F. Kern, Govindjee Govindjee, Johannes Messinger

**Affiliations:** 1 Department of Chemistry, Chemical Biological Centre, Umeå University, 90187 Umeå, Sweden; 2 Molecular Biophysics and Integrated Bioimaging Division, Lawrence Berkeley National Laboratory, Berkeley, CA 94720 USA; 3 Department of Plant Biology, Department of Biochemistry and Center of Biophysics & Quantitative Biology, University of Illinois at Urbana-Champaign, Urbana, IL 61801 USA; 4 Molecular Biomimetics, Department of Chemistry – Ångström, Uppsala University, 75120 Uppsala, Sweden

**Keywords:** Photosynthesis, Function of Photosystem II, Primary photochemistry, Oxygen evolution, Mechanism of water oxidation, Educational review

## Abstract

Photosynthetic water oxidation by Photosystem II (PSII) is a fascinating process because it sustains life on Earth and serves as a blue print for scalable synthetic catalysts required for renewable energy applications. The biophysical, computational, and structural description of this process, which started more than 50 years ago, has made tremendous progress over the past two decades, with its high-resolution crystal structures being available not only of the dark-stable state of PSII, but of all the semi-stable reaction intermediates and even some transient states. Here, we summarize the current knowledge on PSII with emphasis on the basic principles that govern the conversion of light energy to chemical energy in PSII, as well as on the illustration of the molecular structures that enable these reactions. The important remaining questions regarding the mechanism of biological water oxidation are highlighted, and one possible pathway for this fundamental reaction is described at a molecular level.

## Photosynthetic light reactions and the role of photosystem II

**Fig. 1:**
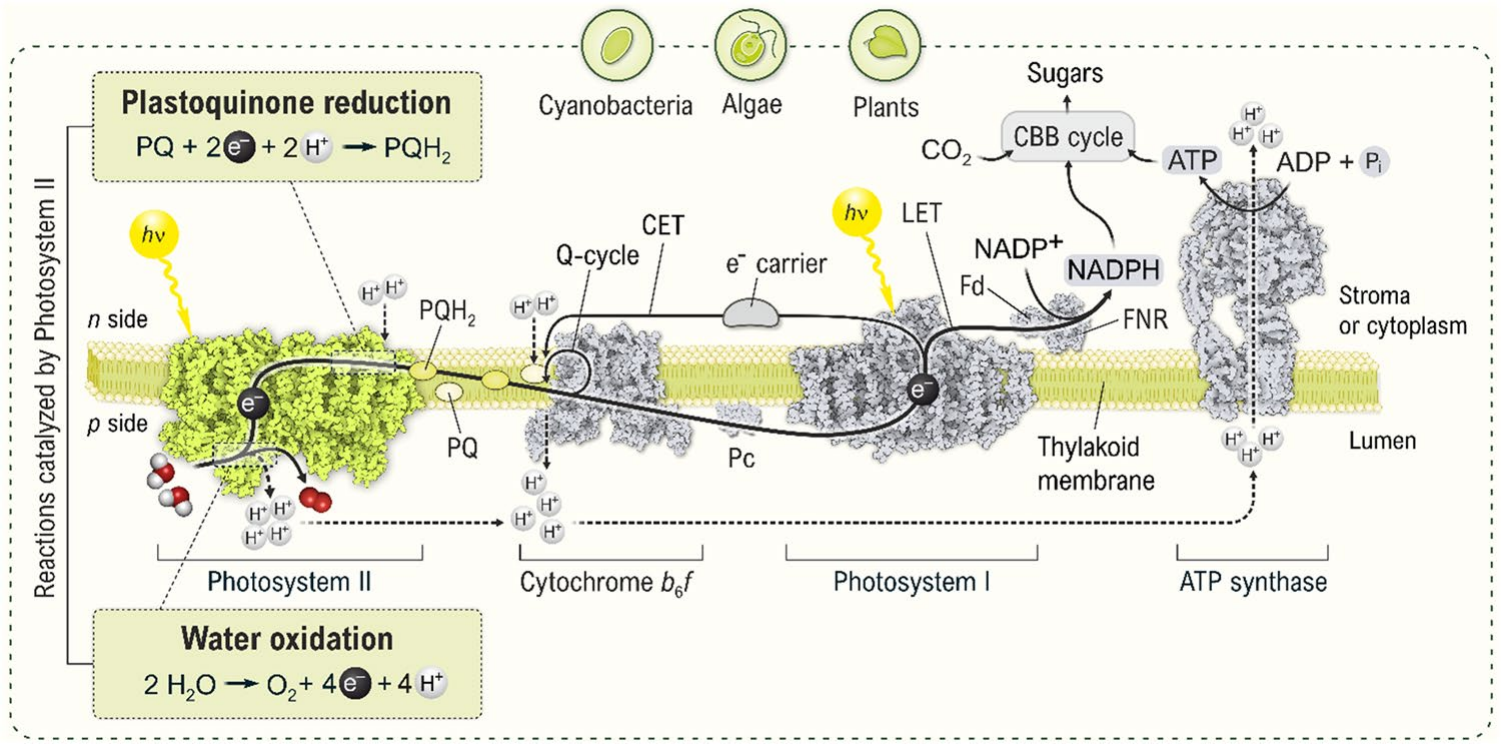
Schematic representation of the light-induced electron (solid arrows) and proton (dashed arrows) transfer reactions driven by the photosynthetic protein complexes embedded in the thylakoid membrane of oxygenic organisms (plants, algae, and cyanobacteria). Whereas in cyanobacteria the thylakoid membrane forms one continuous membrane system within the *cytoplasm*, in algae and plants this membrane is located in a special organelle, the *chloroplast*. The photosynthetic pigment-protein complexes, PSII, Cyt *b*_6_*f*, and PSI bind the redox cofactors (not shown) needed to drive the *linear electron transfer* (LET; long bold arrow) from H_2_O to the oxidized form of nicotinamide-adenine dinucleotide phosphate (NADP^+^) (Govindjee et al. 2017). The diagram also shows the *cyclic electron transfer* (CET) around PSI toward PQ and Cyt *b*_6_*f* and then back to PSI (Joliot et al. 2006), which occurs under specific conditions, and by the *Q-cycle* in Cyt *b*_6_*f* (Cramer et al. 2011; Tacchino et al. 2019). These two cycles increase the number of protons pumped across the membrane per transferred electron, thus resulting in an increased generation of ATP by the ATP synthase and a decreased production of NADPH. Data indicate that the balance between LET and CET is regulated by dynamic thylakoid stacking (Wood et al. 2018). PSII plays an indispensable role by catalyzing the oxidation of water and the reduction of plastoquinone (PQ), and thus providing both the electrons and lumenal protons that drive NADPH and ATP synthesis (Davis et al. 2016). The proton transfer steps are depicted by black dashed arrows. Importantly, the photosynthetic complexes do not occur in thylakoid membranes in a 1:1:1:1 ratio and are not lined up as shown in our simplified diagram here (Danielsson et al. 2004; Koochak et al. 2019; Rantala et al. 2020). PQ/PQH_2_, mobile oxidized/reduced plastoquinone molecules performing the electron transfer from PSII to Cyt *b*_6_*f* and contributing to the proton transfer into the lumen; *Pc*, plastocyanin, a mobile copper-containing protein conducting the electron transfer between Cyt *b*_6_*f* and PSI (note that cyanobacteria mainly use the iron-containing protein Cyt *c*_6_, also known as Cyt *c*_553_ (Zhang et al. 1992), and only under certain conditions Pc is used to transfer electrons from Cyt *b*_6_*f* to PSI); *Fd*, ferredoxin; *FNR*, ferredoxin-NADP^+^ reductase; *CBB cycle*, Calvin-Benson-Bassham cycle. Graphical representations of the complexes were generated using coordinates of the following PDB codes: 1AG6, 1VF5, 3W5U, 4Y28, 5XNL, and 6B8H. The figure is adapted from Agrisera Educational Poster 5 (Shevela et al. 2021a). Reproduced with permission of Agrisera AB (Sweden)

All aerobic life on Earth is totally dependent on a fundamental biological process, the *oxygenic photosynthesis*, which utilizes the energy of sunlight to produce organic matter from water (H_2_O) and carbon dioxide (CO_2_), and releases molecular oxygen (O_2_) into the atmosphere. This process occurs both in prokaryotic (cyanobacteria) and eukaryotic (algae and higher plants) organisms and can conceptually be divided into the *light reactions* and *the carbon fixation* (‘dark reactions’). The light reactions are performed by a set of four transmembrane protein complexes, which together with lipids form the *thylakoid membrane*. Three of the four protein complexes are functionally connected by mobile electron carriers (Fig. 1). The light reactions begin with the absorption of light (*photons*) by the antenna system, which delivers the excitation energy gained to two of the four protein complexes, *Photosystem I (PSI)* and *Photosystem II (PSII)*, which are multimeric pigment-protein complexes, each containing a central region referred to as the *Reaction Center (RC)*. The RCs trap the excitation energy using special photoactive pigment molecules, which perform the *primary photochemistry* that results in the formation of one positively and one negatively charged molecule, and thereby leads to the conversion of light energy into chemical energy. The separated charges are subsequently stabilized by a sequence of electron transfer steps, and finally utilized to drive redox chemistry. By the interplay of the four protein complexes, which couples the light reactions of PSII and PSI (Fig. 1), the linear electron transfer chain of photosynthesis, known as *Z-scheme* (reviewed in Govindjee et al. 2017), bridges a large energy gap and thereby allows the transfer of electrons from H_2_O to the oxidized form of nicotinamide adenine dinucleotide phosphate (NADP^+^). Importantly, this light-driven electron transfer is intertwined with the transfer of protons from one side of the thylakoid membrane [*n* (negative) side; *stroma* in plants and algae or *cytoplasm* in cyanobacteria] to the other [*p* (positive) side or *lumen*]. The lumen is an enclosed space (Kieselbach and Schröder 2003) and the resulting imbalance in proton concentration and charge distribution resembles a ‘biological battery.’ The energy of this battery is utilized by the enzyme *Adenosine triphosphate* (ATP) *synthase* for making ATP from adenosine diphosphate (ADP) and inorganic phosphate (reviewed in Allen 2003; Junge and Nelson 2015). Thus, the light reactions of oxygenic photosynthesis lead to the storage of solar energy in the chemical bonds of NADPH and ATP (see Fig. 1 and its legend). This chemical energy powers the carbon fixation reactions, *i.e.*, the conversion of CO_2_ to carbohydrates. This process is also known as the *Calvin-Benson-Bassham cycle* (CBB cycle), and is catalyzed by the enzyme *Rubisco* (ribulose-1,5-bisphosphate carboxylase/oxygenase) in the stroma/cytoplasm (reviewed in Bassham 2005; Benson 2005; Andersson and Backlund 2008; Sharkey 2019; Gurrieri et al. 2021). In this way, the absorbed solar energy is converted and stored more permanently, and the initial building blocks for all kinds of biomass are formed.

The linear electron transport chain starts with PSII, which catalyzes the light-driven extraction of electrons from water, leading to the production of O_2_ at its electron donor side, and the reduction of plastoquinone (PQ) to plastoquinol (PQH_2_) on its electron acceptor side. The overall reaction can thus be described as

1 $$2{\text{H}}_{{2}} {\text{O}} + 2{\text{PQ}} + 4{\text{H}}_{stroma/cytoplasm}^{ + } \xrightarrow{hv} {\text{O}}_{2} + 2{\text{PQH}}_{2} + 4{\text{H}}_{lumen}^{ + }$$

This equation also signifies that the oxidation of two water molecules results in the release of four protons into the lumen (the ‘*p*’ side), while four protons are taken up from the stromal/cytoplasmic (the ‘*n*’) side of the thylakoid membrane during the formation of two molecules of PQH_2_ (see Fig. 1). Oxidation of these two PQH_2_ molecules by the *Cytochrome* (Cyt) *b*_6_*f* complex (Malone et al. 2021; Sarewicz et al. 2021) leads to the release of these four protons into the lumen (in addition to four protons from water oxidation). Furthermore, protons can be pumped by the so-called *Q-cycle* taking place within Cyt *b*_6_*f* (see Fig. 1 and its legend), and by *cyclic electron transfer* (CET), in which electrons are transferred back from PSI to PQ and then to Cyt *b*_6_*f*. These cycles are engaged under specific physiological circumstances, so that a large number of protons become available in the lumen for ATP production (Nawrocki et al. 2019). Note that ATP synthesis is powered by both a difference in proton concentration between both sides of the thylakoid membrane (ΔpH), and by the electrical potential difference (ΔΨ) between both sides that is created by the directional charge separations in PSI and PSII (Lyu and Lazár 2017), because both processes create positive charges near the lumen (*p* side) and negative charges near the stroma/cytoplasm (*n* side). Together, they form the *proton motive force* (*pmf*), which can be regulated additionally by several ion (Cl^−^, K^+^, Na^+^, Mg^2+^, Ca^2+^) pumps/channels (reviwed in Mitchell 2011; Junge and Nelson 2015; Armbruster et al. 2017; Spetea et al. 2017; for further details, see Dukic et al. 2019). Thus, the chemical reactions performed by PSII contribute significantly to the formation of the *pmf* (charging the photosynthetic battery) that drives ATP synthesis.

Further reading: Junge and Nelson (2015), Govindjee et al. (2017), Spetea et al. (2017).

## Photosystem II and its impact on earth’s atmosphere and life

**Fig. 2:**
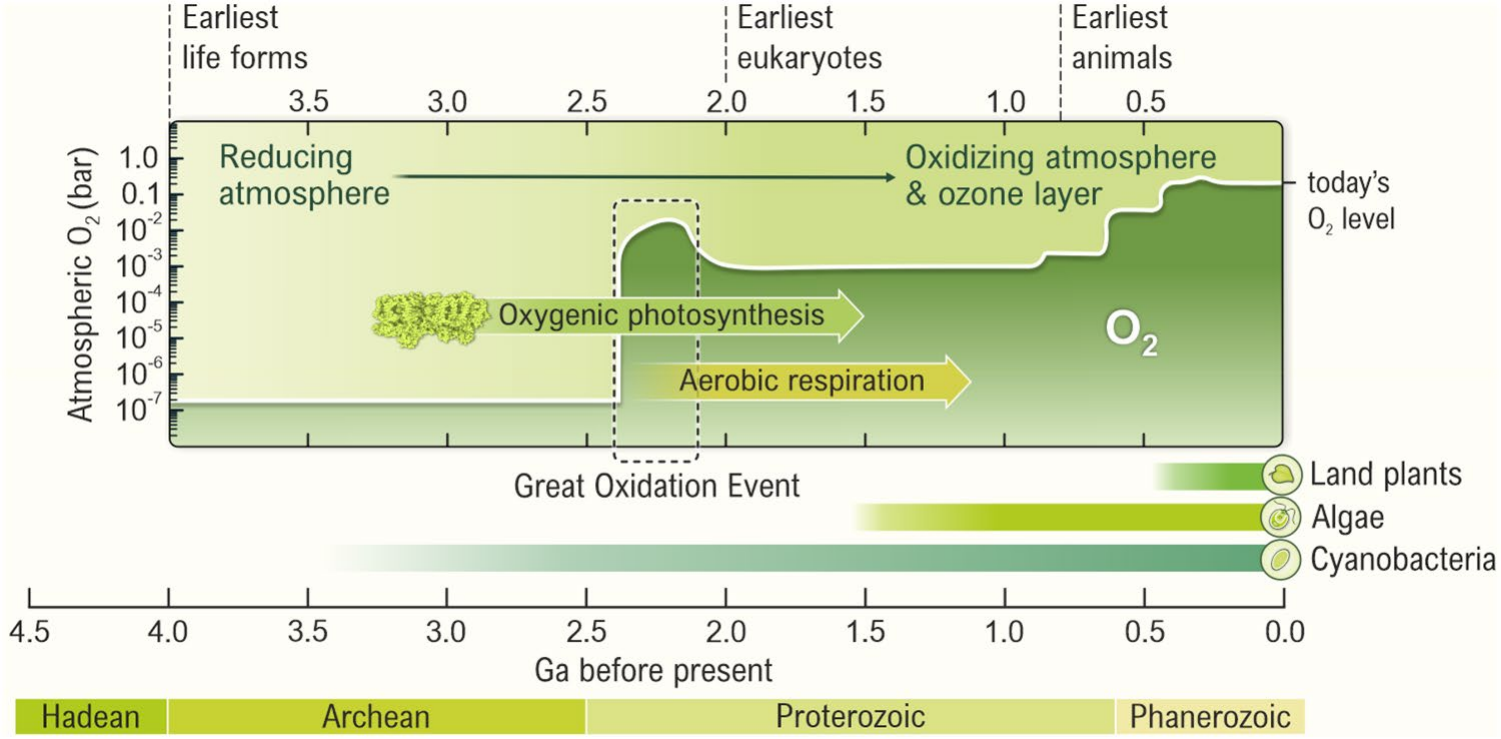
The impact of photosystem II (PSII) on changing the concentration of O_2_ in the Earth’s atmosphere and the evolution of life as a function of geological time in billions of years (*giga* years, Ga; 1 Ga = 10^9^ years). Most researchers agree that between 2.4 and 2.1 billion years ago, there was a large and ‘sudden’ increase of the O_2_ level in the atmosphere (note the logarithmic scale for O_2_ concentration), known as the *Great Oxidation Event* (GOE) (Kump 2008; Schopf 2011; Blaustein 2016; Luo et al. 2016; Gumsley et al. 2017; Catling and Zahnle 2020). However, the time point when the oxygenic photosynthesis evolved is still under discussion. Current estimates range from ~ 3.5 billion years ago (or even earlier) to the time points close to the GOE (Farquhar et al. 2011; Planavsky et al. 2014; Satkoski et al. 2015; Schirrmeister et al. 2015; Fischer et al. 2016; Hamilton et al. 2016; Soo et al. 2017; Garcia-Pichel et al. 2019; Catling and Zahnle 2020; Fournier et al. 2021; Oliver et al. 2021). The unique advent of O_2_ evolution was, undoubtedly, the *biological Big Bang* for the evolution of the whole biosphere, since it created the requisite background for the development and sustenance of the aerobic metabolism that is the energetic basis for all of the more-advanced forms of life (Lane 2004; Falkowski 2006; Barber 2008). Correlation between the estimated changes in atmospheric O_2_ level (logarithmic scale) and the indicated evolutionary events on Earth are based on numerous publications (Kump 2008; Hohmann-Marriott and Blankenship 2011; Blaustein 2016; Fischer et al. 2016; Catling and Zahnle 2020; Oliver et al. 2021). Only some selected events of evolutionary diversification and the origin of some organisms are shown in the diagram. The figure is adapted from Agrisera Educational Poster 5 (Shevela et al. 2021a). Reproduced with permission of Agrisera AB (Sweden)

While the first life forms on Earth developed on chemical energy provided possibly by thermal deep ocean vents in the form of gases such as molecular hydrogen (H_2_) and methane (CH_4_) or by mineral surfaces (Knoll and Nowak 2017; Konhauser et al. 2017; Catling and Zahnle 2020), today the energy for most life on Earth is ultimately provided by the Sun in the form of quanta of electromagnetic radiation, *photons* (or simply sunlight). More than 3 billion years ago, the first photosynthetic organisms ‘learned’ how to capture photons and convert their energy into chemical energy for further use. The emergence of PSII had a tremendous effect on the process of evolution on our Earth, since it allowed some photosynthetic organisms to utilize the abundant water as an electron and proton source for photosynthetic CO_2_ reduction to energy-rich molecules and biomass (see Fig. 2 and its legend). This significantly increased the chemical energy input into the biosphere, and enriched the atmosphere with O_2_ (the by-product of the water-splitting reaction), while reducing the level of CO_2_ and thus its greenhouse effect (Berner 2006; Nisbet and Fowler 2011).

Most studies suggest that the oxygenic photosynthesis first evolved at the level of ancient organisms closely related to today’s cyanobacteria, *i.e.*, after the development of anoxygenic photosynthetic bacteria (Blankenship et al. 2007; Hohmann-Marriott and Blankenship 2011; Schopf 2014; Schirrmeister et al. 2015; Soo et al. 2017; Garcia-Pichel et al. 2019; Fournier et al. 2021; Sánchez-Baracaldo et al. 2021). However, two recent studies propose that the emergence of photosynthetic water oxidation may have happened closer to the origin of life, and that PSII might be among the oldest of the enzymes (Sánchez-Baracaldo and Cardona 2020; Oliver et al. 2021). Thus, according to these results, anoxygenic photosynthesis would have developed from oxygenic organisms.

The above-mentioned increased O_2_ concentration also affected the lithosphere by promoting the formation of mineral oxides. While the rising O_2_ level was toxic to many species at the time, it eventually allowed higher life forms to develop since 10–15 times more energy could be extracted from organic molecules by respiration as compared to anaerobic processes such as fermentation (Lane 2004; Payne et al. 2011; Peschek et al. 2011). Importantly, the O_2_ in the atmosphere was also the basis for the formation of the ozone (O_3_) layer that reduced the level of UV radiation at the surface of Earth and thereby facilitated the spread of life from the oceans onto the land (Olson and Blankenship 2004; Segura et al. 2004; Kump 2008; Thomassot et al. 2015). Further evolution and development of oxygenic photosynthetic organisms produced enormous amounts of biomass, which was partly transformed into fossil fuels (coal, oil, and natural gas), and created today’s aerobic atmosphere with ~ 21% O_2_.

Over the past 3 billion years, enzymatic activity of PSII has produced the oxygen we breathe, and has thus been the essential driver in the evolution that has transformed Earth and life on it to the way we experience it today (Vinyard et al. 2013; Junge 2019). The interesting question as to how the ability to extract electrons and protons from H_2_O using the energy of sunlight evolved is still under investigation; see the legend of Fig. 2 and several papers on this topic (Olson 2006; Blankenship 2010; Hohmann-Marriott and Blankenship 2011; Sousa et al. 2013; Fischer et al. 2016; Khadka et al. 2017; Soo et al. 2017; Cardona 2019; Cardona and Rutherford 2019; Oliver et al. 2021).

Further﻿ reading: Catling and Zahnle (2020), Fournier et al. (2021), Blankenship (2021).

## Overview of the structure and function of photosystem II

**Fig. 3:**
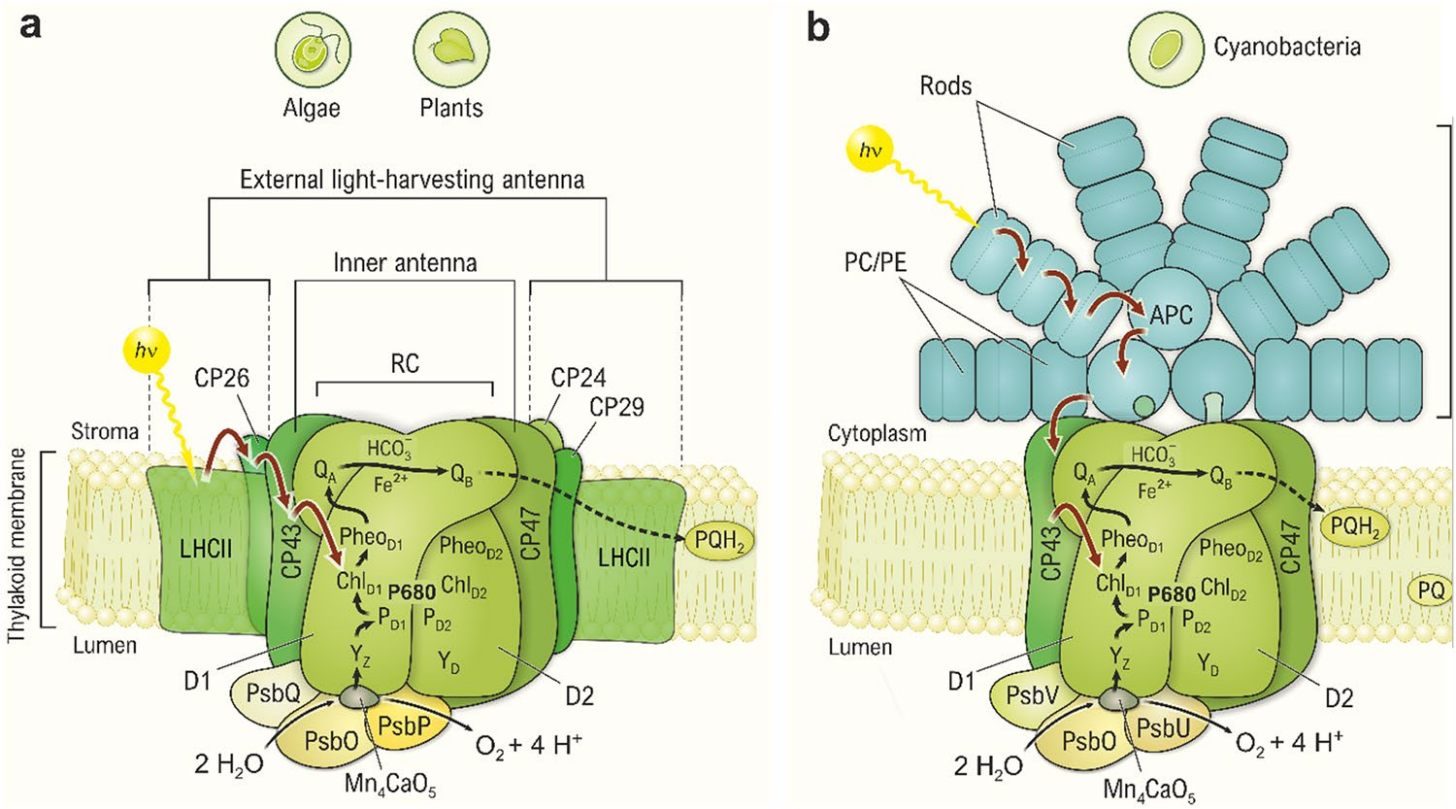
Simplified schematic representation of the main proteins and electron transfer cofactors in PSII of higher plants and green algae (**a**) and cyanobacteria (**b**) with their attached antenna systems. Functionally, each PSII monomer can be divided into the PSII core and a connected light-harvesting antenna. At the center of the PSII core is the RC that is formed by the D1 and D2 proteins, which harbor all the redox cofactors needed for photochemical primary charge separation (Fig. 6) and the subsequent electron transfer steps (indicated by black arrows), including the reactions for water oxidation (Figs. 7 and 8) and plastoquinone reduction (Fig. 9). In addition, the PSII core comprises the inner antenna proteins CP43 and CP47 as well as 13 other intrinsic and 3 extrinsic protein subunits. The cofactors of the PSII RC include the Mn_4_CaO_5_ cluster, the tyrosine Y_Z_, a Chl dimer (P_D1_ and P_D2_), two monomeric Chls (Chl_D1_ and Chl_D2_), a Pheo_D1_, and two plastoquinone molecules (permanently bound Q_A_ and mobile Q_B_). The classical term for the primary electron donor, *‘P680’*, which is also used in the introductory text, is ill-defined at the molecular level, but it is best to include, in it, P_D1_/P_D2_, Chl_D1_, and Chl_D2_ (Durrant et al. 1995). The number ‘680’ originates from the wavelength of the absorption maxima of these Chls in the red region (Rabinowitch and Govindjee 1965). Note that except for Q_A_, all the active cofactors are located on the ‘left’ D1-branch of the apparently symmetric cofactor arrangement. The other cofactors of the D2-branch (such as tyrosine Y_D_, Pheo_D2_) are thought to have protective roles in PSII, since they are also redox active, and only capable of donating electrons to P680^•+^, when the electron transfer through the Mn_4_CaO_5_ cluster/Y_Z_ path is inactive. The non-heme iron (Fe^2+^), located between Q_A_ and Q_B_, normally does not directly take part in the electron transfer, but plays an important role by binding a bicarbonate ion (HCO_3_^−^), which participates in the protonation of Q_B_^−^ (reviewed in Müh et al. 2012; Shevela et al. 2012; Müh and Zouni 2013; also, see Umena et al. 2011; Brinkert et al. 2016). If Q_A_^−^ cannot be oxidized by Q_B_, for example, under high light stress or when the carbon fixation cycle is limiting, the bicarbonate ion dissociates, which changes the redox potential of Q_A_/Q_A_^−^ and, thereby, protects PSII by minimizing charge recombination (Brinkert et al. 2016; Shevela et al. 2020). CP43 and CP47 are protein complexes binding several Chl *a* molecules, which function as core (or ‘inner’) antenna of PSII. PsbQ (17 kDa), PsbO (33 kDa), and PsbP (23 kDa) are extrinsic proteins in higher plants (**a**), while in cyanobacteria, two of these proteins, PsbQ and PsbP, are substituted by PsbV (or Cyt *c*550; 17 kDa) and PsbU (12 kDa) (**b**), respectively (Bricker et al. 2012; Ifuku and Noguchi 2016). Both in higher plants and cyanobacteria, these proteins stabilize the Mn_4_CaO_5_ cluster and optimize its water-oxidizing activity. While the structures and functions of the PSII RC in all oxygenic organisms are very similar, the structures of the external (outer) light-harvesting complexes (LHCs) in these photosynthetic organisms are significantly different (also see Fig. 4). Higher plants and green algae capture sunlight utilizing the major membrane-integral LHCII and some minor chlorophyll-protein complexes, such as CP24 (24 kDa), CP26 (26 kDa), and CP29 (29 kDa) (Croce and van Amerongen 2020; Müh and Zouni 2020). However, the major LHCs of PSII in cyanobacteria (as well as in red and glaucophyte algae) are the PBS that in contrast to the LHCIIs are not membrane-integral, but attached to the cytoplasmic surface of PSII RCs (Green 2019; Sui 2021). PBSs are made of pigments, the *phycobilins* (PB), and the proteins, the phycobiliproteins; they are the *phycocyanins* (PC), the *allophycocyanins* (APC), and the *phycoerythrins* (PE). Light absorption  (photons, *hν*) is indicated by yellow arrows, while excitation energy transfer is indicated by red arrows, whereas the electron transfer is indicated by black arrows. The figure is adapted from Agrisera Educational Poster 5 (Shevela et al. 2021a). Reproduced with permission of Agrisera AB (Sweden)

**Fig. 4:**
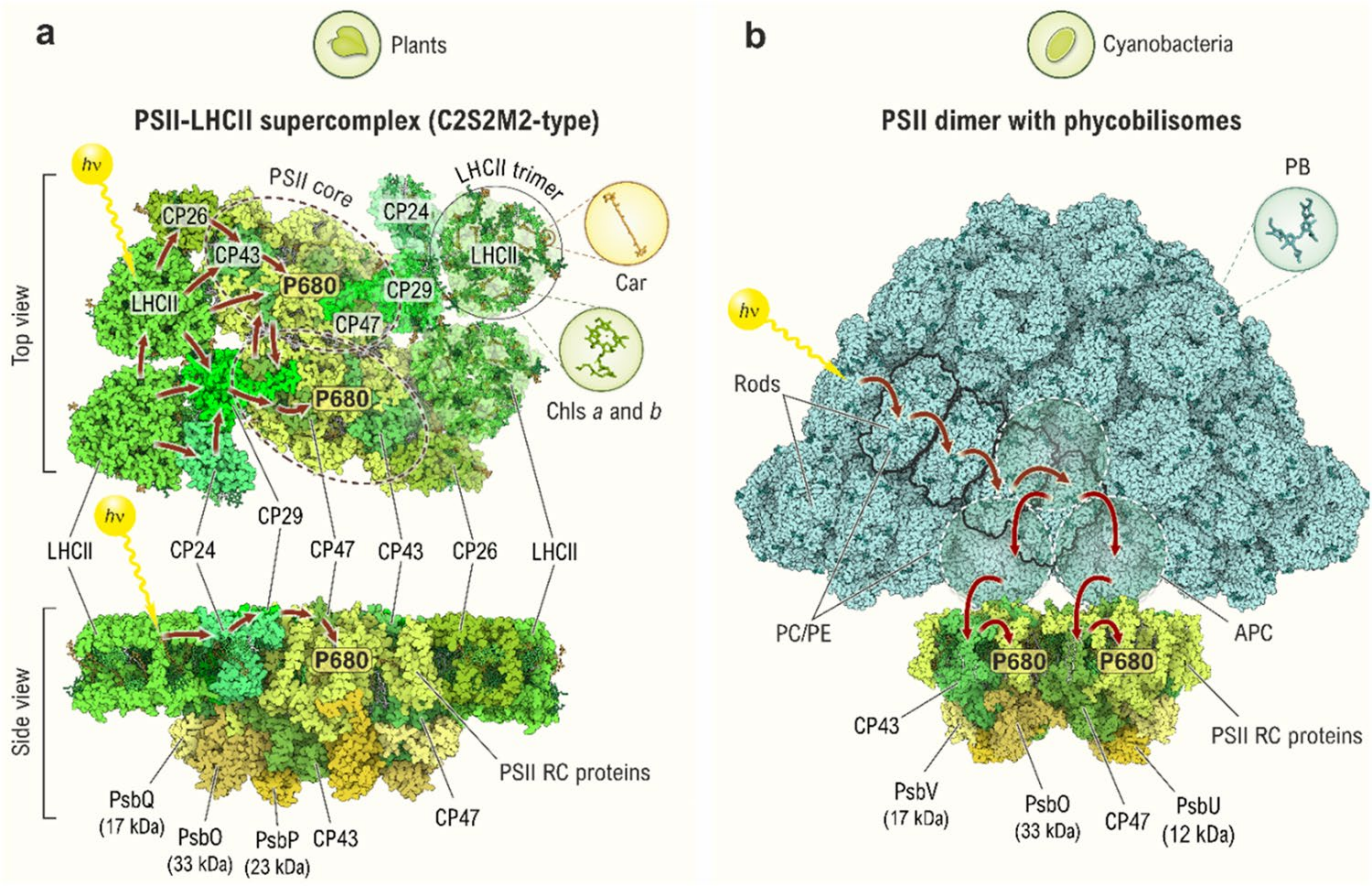
Overall organization of PSII dimers with attached light-harvesting antenna systems in plants and cyanobacteria. **a** Plant PSII-LHCII supercomplex (C2S2M2-type; *i*.*e*., a complex, which has two PSII cores (C), two strongly (S), and two moderately (M) bound LHCII trimers). We show here its subunit composition as viewed from the stromal side (*top*) and along the membrane plane (*bottom*). Note that the number and the position of LHCII trimers as well as of the other light-harvesting subunits may vary between plants and green algae (Su et al. 2017; Shen et al. 2019; Sheng et al. 2019; Croce and van Amerongen 2020). The minor antenna proteins (CP24, CP26, CP29), and major antenna complexes (LHCII trimers; labeled as LHCII), as well as the inner antenna proteins (CP43 and CP47) and extrinsic proteins (PsbQ, PsbO, and PsbP) are colored individually, whereas other PSII core proteins (see Fig. 5a) are colored collectively in yellow. The approximate boundary of each PSII core is indicated by dashed circles. The pigments of the light-harvesting complexes (LHCs) in plants include Chls *a* and *b* and Cars, which are bound in specific geometric arrangements (Müh and Zouni 2020), as shown in one of the LHCII trimers marked by a circle. The LHCII is a trimer, with each monomer harboring eight molecules of Chl *a*, six of Chl *b,* and four of Cars. **b** A side-on view (perpendicular to the membrane normal) of a cyanobacterial PSII dimer with PBS anchored to the cytoplasmic side of the PSII complex. PBSs are formed by the *phycobiliproteins*: APC, organized into core cylinders (two in the base and one on the top, as indicated by white dashed circles), and *phycocyanins* (PC) and, in some cyanobacteria, *phycoerythrins* (PE) organized into peripheral rods (Sui 2021). All phycobiliproteins contain covalently bound pigment, PB. In both panels, red arrows indicate possible direction of the excitation energy transfer toward the RC Chls *a*, denoted as P680. The PSII models were generated with *The Protein Imager* (Tomasello et al. 2020) using the coordinates deposited at the following PDB IDs: 5XNL (for PSII-LHCII supercomplex), 6W1O (for cyanobacterial PSII dimer; for the first high-resolution X-ray structure, see 3WU2), and 6KGX (for PBS from the red alga *Porphyridium purpureum*, which is currently the only available entire PBS structure (Ma et al. 2020); however, the algal PBS structure is thought to be very similar to PBS in cyanobacteria). For other abbreviations, see the legend of Fig. 3. The figure is adapted from Agrisera Educational Poster 5 (Shevela et al. 2021a). Reproduced with permission of Agrisera AB (Sweden)

The cores of all PSII complexes, from cyanobacteria, green and red algae, and plants, are remarkably similar both with regard to their structure and function. Simplified schematic pictures of PSII from higher plants and cyanobacteria with their redox-active cofactors and associated antenna systems are shown in Fig. 3, while Fig. 4 presents the corresponding structural models derived by x-ray crystallography or cryo electron microscopy (cryo-EM).

PSII is a large multimeric pigment-protein complex that occurs in the thylakoid membranes of these organisms as a dimer (Fig. 4) with a total molecular weight of ∼700 kDa. Since the two monomers of each dimer are functionally largely independent from each other, we show only one monomer in the schematic of PSII (Fig. 3).

For each PSII monomer, all redox-active cofactors required for photochemical charge separation, water oxidation, and the reduction of PQ, are bound by the D1 (PsbA) and D2 (PsbD) proteins that form a heterodimer in the center of PSII (Fig. 3). This D1/D2 heterodimer, also referred to as the RC, is flanked by two inner, chlorophyll (Chl) binding proteins, CP43 and CP47 (Vinyard et al. 2013; Shen 2015; Müh and Zouni 2020). In addition, the membrane intrinsic Cyt *b*_559_, comprised of the two subunits PsbE and PsbF, is an indispensable component of PSII, but its role is not yet fully defined (Cramer and Zakharov 2022). Water oxidation takes place at the tetra-manganese calcium penta-oxygen cluster (Mn_4_CaO_5_), which is coordinated by conserved amino acid residues mostly provided by the D1 protein, but the CP43 protein also provides one ligand (for the first high-resolution structure of the PSII, see Umena et al. 2011; Suga et al. 2015). The extrinsic proteins, which differ between plants/algae (PsbO, PsbQ, PsbP) and cyanobacteria (PsbO, PsbU, PsbV), are important for the stability of the Mn_4_CaO_5_ cluster (reviewed in Roose et al. 2016; Ifuku and Nagao 2021). In addition, at least another 11 membrane intrinsic subunits are found in highly active PSII core preparations, bringing the minimum number of proteins required for full activity to at least 20. We refer to this functional unit as PSII core. For additional small proteins and their possible functions, see Shi and Schröder (2004).

The PSII core is connected to an additional outer antenna, the light-harvesting complex (LHC), which differs significantly between the prokaryotic and the eukaryotic photosynthetic organisms. In higher plants and green algae, this outer antenna complex is made of membrane-integral pigment-protein complexes collectively referred to as the *light-harvesting complex II* (LHCII). However, in cyanobacteria (as well as in red algae), the LHC is known as the *phycobilisome* (PBS) and is attached to the cytoplasmic side of PSII. In both cases, the antenna consists of protein complexes that bind the light-absorbing molecules (*pigments*). Plants and algae employ Chls and some other accessory pigments, such as *carotenoids* (Cars), while in cyanobacteria, *phycobilins* (PB) are the major light absorbers. All the pigments capture photons and transfer the excitation energy to the PSII RC, where the primary charge separation and chemical reactions take place. The large variety of pigments and outer antenna systems allow the various organisms to adapt to different light intensities and light quality, and the detachment of the outer antenna from PSII is one of many regulatory mechanisms that help protect PSII from damage in bright light (reviewed in Krieger-Liszkay et al. 2008; Tikkanen and Aro 2012; Vass 2012; Derks et al. 2015). The overall reaction of PSII can be conceptually divided into three processes:

*1. Light Absorption and Excitation Energy Transfer* (EET) by the antenna system of PSII (ultra-fast process).

*2. Charge Separation* (very fast) *and Stabilization* (fast) at the PSII RC.

*3. Chemical Reactions* (slow): H_2_O oxidation and PQ reduction, as shown below:

2 $$2\!{\text{ H}}_{2} {\text{O }}\xrightarrow{h\nu } {\text{O}}_{2 } + 4 {\text{e}}^{ - } + 4 {\text{H}}_{lumen}^{ + } \,\left( {{\text{at the lumenal side of PSII}},{\text{ the}}\,p\,{\text{side}}} \right)$$

3 $$2{\text{PQ}} + 4 {\text{e}}^{ - } + 4\!\!{\text{ H}}_{stroma}^{ + } { }\xrightarrow{h\nu } 2\!\!{\text{ PQH}}_{2 } \,({\text{at the stromal}}/{\text{cytoplasmic side of PSII}},{\text{ the}}\,n\,{\text{side}})$$

The time scales of these three steps and energetic implications are discussed below in a general way in order to best convey the concepts, while for details we refer to the subsequent sections.

**Fig. 5:**
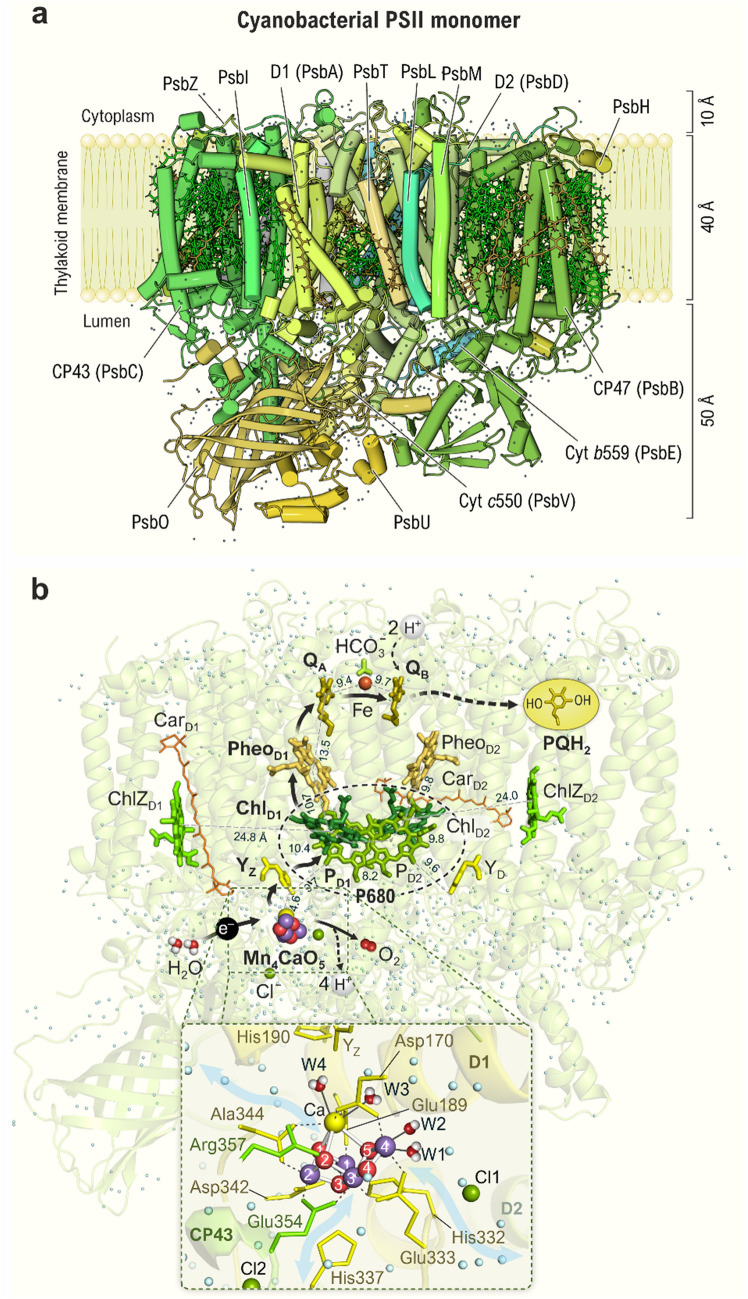
Structure of the cyanobacterial PSII complex (monomer without phycobilisomes) and arrangement of its central redox cofactors as seen along the thylakoid membrane with the lumen at the bottom and the cytoplasm at the top. **a** Structure of the PSII monomer embedded into a schematically drawn thylakoid membrane. Protein subunits (with helices visualized as cylindrical tubes) are colored and labeled individually in the figure. Chls and Cars are shown in green and orange, respectively. For the sake of clarity, some small protein subunits of PSII (Shi et al. 2012) have been omitted. Note that PSII extends to ~ 10 Å into the cytoplasm and up to ~ 50 Å into the lumen. **b** Arrangement of the electron transfer cofactors in the RC of PSII. The cofactors are visualized in color and the protein scaffold is shown as a light-green background. The electron transfer direction is indicated by arrows. During primary photochemistry (see Fig. 6a), light energy is converted into chemical energy by transferring an electron from the primary electron donor P680 (Chl *a* molecules P_D1_, P_D2_, Chl_D1_, and Chl_D2_) to the primary electron acceptor Pheo_D1_. From Pheo_D1_ the electron is transferred first to the plastoquinone Q_A_, and then to the plastoquinone Q_B_. We note that the latter transfer is accelerated by HCO_3_^−^ (bound to the Fe^2+^), which is known to play an essential role in this process by facilitating proton transfer to the reduced Q_B_ (Wydrzynski and Govindjee 1975; Brinkert et al. 2016; also reviewed in Govindjee and Van Rensen 1993; Shevela et al. 2012; Müh and Zouni 2013). Each Q_B_ molecule accepts, in sequential steps, two electrons. The fully reduced Q_B_ picks up two protons (see Fig. 9), thus forming plastoquinol (PQH_2_), which is then released from the Q_B_ site (shown by dashed arrow) into the lipid membrane (Müh et al. 2012; Van Eerden et al. 2017). Y_Z_ (Tyr_Z_) and Y_D_ (Tyr_D_) are redox-active tyrosine residues, of which Y_Z_ acts as an electron carrier between the catalytic site of water oxidation (Mn_4_CaO_5_ cluster) and the P680. Distances between some cofactors, given in Å, are shown in the diagram. The bottom of panel **b** shows a zoomed view of the Mn_4_CaO_5_ cluster and its protein environment. The surrounding protein residues of the D1 protein are shown in yellow, and those from CP43 in green. The three suggested water and proton channels (Hussein et al. 2021) that connect the Mn_4_CaO_5_ cluster with the lumen are indicated by broad light-blue arrows. Mn atoms (purple spheres) are numbered 1–4, bridging oxygen atoms (red spheres) as 1–5, and metal bound water molecules as W1–W4. Note that the protons on W1-W4 are shown only for illustrative purposes; they are not resolved in available crystallographic data. All other water molecules, found in the crystal structure, are shown as cyan spheres. Ca^2+^ and Cl^−^ ions are shown as yellow and green spheres, respectively. The structures were generated by using the coordinates from PDB code 6W1O (for the first high-resolution X-ray structure of PSII, see 3WU2). The figure is adapted from Agrisera Educational Poster 5 (Shevela et al. 2021a). Reproduced with permission of Agrisera AB (Sweden)

The *absorption* of photons occurs mostly by the LHCs, which is shown in Fig. 3 by *hν* and yellow arrows. This process occurs at the femtosecond time scale (1 fs = 10^−15^ s) and leads to the generation of excited electronic singlet states in the pigments (Chls or PBs). EET (red arrows in Figs. 3 and 4) occurs via the pigments of the LHCs and of the inner antenna proteins. Eventually, the excitation energy reaches the primary electron donor of the PSII RC, which is an ensemble of four Chls *a* (Chl_D1_/P_D1_/P_D2_/Chl_D2_; see Figs. 3–5) known as *P680* since its long-wavelength absorption maximum is at ~ 680 nm (while its short-wavelength absorption maximum is at ~ 440 nm). When this happens, P680 reaches its excited singlet state (^1^P680*), from which it then transfers the excited electron to the nearby primary electron acceptor, pheophytin (Pheo_D1_; see Figs. 3 and 5). This electron transfer constitutes the *primary charge separation* and leads to the formation of the radical pair P680^•+^Pheo_D1_^•–^. This step occurs within ~ 3 ps (Wasielewski et al. 1989; Greenfield et al. 1997), *i.e.*, more than 1000 times slower than light absorption and EET (1 ps = 1000 fs). It is noteworthy that the cation radical P680^•+^ has one of the highest known oxidizing potentials (~ + 1.25 V) in biology (Diner and Rappaport 2002; Ishikita et al. 2005; Rappaport and Diner 2008; Renger 2012b).

**Fig. 6:**
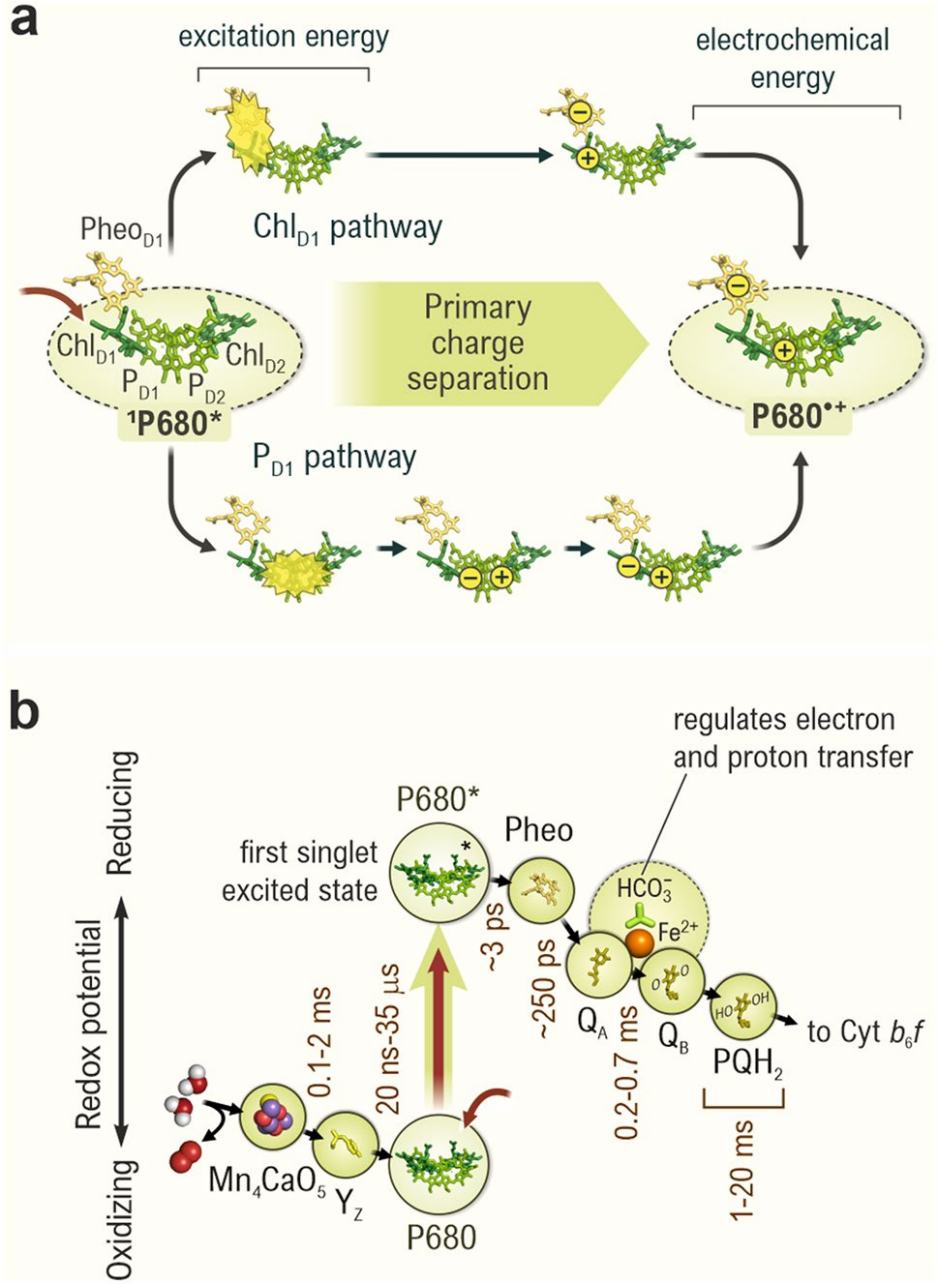
Conversion of light energy to chemical energy by photosystem II (PSII). **a** Simplified schematic representation of the primary charge separation in PSII. Excitation energy (shown by red curved arrow) reaches the photoactive RC-Chl *a* molecules (Chl_D1_, P_D1_, P_D2_, Chl_D2_) and leads to the formation of the singlet excited state of P680, ^1^P680^∗^. As a result, one electron leaves one Chl *a* molecule, thus forming a positively charged chlorophyll radical cation, P680^•+^, and transfers to the Pheo_D1_, which is then reduced and forms a radical anion, Pheo_D1_^•–^. Two alternative pathways exist that can lead to the primary charge-separated state P680^•+^ Pheo_D1_^•–^ (Mamedov et al. 2015; Romero et al. 2017). One pathway involves excitation and charge separation from Chl_D1_ to Pheo_D1_ and is known as *Chl*_*D1*_* pathway*. The other pathway proceeds through the pair P_D2_ P_D1_ to Pheo_D1_ and is known as *P*_*D1*_* pathway*. Both pathways may take place in the same PSII RC, and both lead to the formation of the same charge-separated state P680^•+^ (or more precisely P_D1_^•+^) Pheo_D1_^•–^. The protein conformation of the PSII RC has been suggested to define which pathway would dominate. **b** Part of the Z-scheme (reviewed in Govindjee et al. 2017) that describes the redox cofactors of PSII. The vertical scale indicates the equilibrium midpoint redox potential (*E*_m_, at pH 7) of the electron transfer components. Approximate characteristic times (1/k) for the electron transfers are given. The rate of electron transfer between the redox cofactors in PSII must be controlled for the efficient functioning of the system, whereby the distance and relative orientation of the components, determined by the protein scaffolding, are the key factors (see Fig. 5b) (Moser et al. 1992). The phytol tails of Chls and Pheo, as well as the isoprenyl chains of the quinones are not shown in the diagram. Cofactors were generated using coordinates of the following PDB entries: 3ARC, 3WU2, and 6W1O. For abbreviations, see the legends of Figs. 1–4. The figure is adapted from Agrisera Educational Poster 5 (Shevela et al. 2021a). Reproduced with permission of Agrisera AB (Sweden)

To avoid charge recombination, which would convert the electrochemical energy of P680^•+^Pheo_D1_^•–^ into heat instead of storing it in chemical bonds, the primary charge separation needs to be stabilized by subsequent electron transfer steps that increase the distance and reduce the energy difference between the positive and the negative charge. This is realized by transferring the electron from Pheo_D1_^•–^ to another one-electron acceptor (plastoquinone Q_A_), while P680^•+^ is reduced back to P680 by the redox-active protein side chain tyrosine Z (Y_Z_). These two subsequent electron transfer steps occur with characteristic times of 250 ps (1 ps = 10^−12^ s) and 20 ns–35 µs (1 ns = 10^−9^ s; 1 µs = 10^−6^ s), respectively (Fig. 6). This stabilizes the primary charge separation so that even much slower (1–20 ms: 1 ms = 10^−3^ s) chemical reactions of plastoquinone Q_B_ reduction to plastohydroquinone (PQH_2_) and its exchange with another PQ molecule from the PQ pool in the thylakoid membrane, as well as the oxidation of the Mn_4_CaO_5_ cluster and water oxidation can take place with high quantum yield. At this point, about half of the initial chemical energy of the primary charge separation is stored in the chemical bonds of PQH_2_ (Fig. 6b) (Dau and Zaharieva 2009; Shevela et al. 2021b). PSII, like PSI (Golbeck 2006)**,** is thus optimized for a high quantum efficiency at low light intensities rather than the maximum solar-to-chemical energy efficiency. A prime reason for that may be that charge recombination reactions not only waste energy but, in the presence of O_2_, also lead to the formation of reactive oxygen species (ROS) that damage PSII, and thus make energy-intensive repair reactions necessary—a wasteful process (Rutherford et al. 2012; Mattila et al. 2015; Pospíšil 2016; Kale et al. 2017; Weisz et al. 2017).

While the photochemistry creates one electron and one hole in each light-induced charge separation (one-electron chemistry), the reduction of Q_B_ on the electron acceptor side of PSII requires two electrons and two protons (two-electron two-proton chemistry; Eq. 3), and for the oxidation of two water molecules to molecular oxygen, four electrons and four protons need to be removed (four-electron four-proton chemistry; Eq. 2) (Babcock et al. 1989). On the acceptor side, this mismatch is overcome by the tight binding of the Q_B_^•–^ intermediate in the binding pocket until the next charge separation occurs and the next electron and the protons are delivered. On the other hand, Q_B_H_2_ has a very low binding affinity so that it leaves its binding site to be replaced by a new PQ molecule. A completely different solution is used on the electron donor side of PSII. Here, a special cofactor, the Mn_4_CaO_5_ cluster, forms the required link between one and four electron chemistry. As such, the Mn_4_CaO_5_ cluster stores four oxidizing equivalents created by four sequential charge separations. In this process, the bound substrate water molecules are stepwise deprotonated, and only after the fourth electron is removed O_2_ is formed (reviewed, for example, in Debus 1992; McEvoy and Brudvig 2006; Dau et al. 2010; Renger 2012a; Yano and Yachandra 2014; Shen 2015; Cox et al. 2020).

The performance of PSII and the Mn_4_CaO_5_ cluster can be measured in many ways, which is also interesting for comparison to synthetic water oxidation catalysts and solar-to-fuels devices. The efficiency by which PSII performs the primary charge separation and subsequent charge stabilization is the *PSII quantum efficiency*. Under optimal (low) light intensities and appropriate spectral light composition, this process can be as efficient as ~ 90%. However, under high light, many (regulatory) processes vastly reduce this quantum efficiency (van Wijk and van Hasselt 1990). An important different parameter is the solar-to-chemical energy efficiency, by which the chemical energy stored is compared to that contained in photons over the entire solar spectrum. This has been estimated to reach values of up to ~ 16% under optimal conditions (Dau and Zaharieva 2009). The rate at which PSII can split water, *i.e.*, its turnover frequency, is limited by the acceptor side reactions (PQH_2_/PQ exchange) to about 50 O_2_ s^−1^ or 200 electrons s^−1^ (Lee and Whitmarsh 1989; Ananyev and Dismukes 2005). The stability of PSII, or its *turnover number*, is limited by destructive side reactions. PSII is capable of producing ~ 100 000 O_2_ molecules before it needs to be repaired (reviewed in Nixon et al. 2010; Järvi et al. 2015; Theis and Schroda 2016). A special repair mechanism is in place that involves the exchange of the damaged D1 protein, followed by the reassembly of the Mn_4_CaO_5_ cluster (Barber and Andersson 1992; Oliver et al. 2022).

Further reading: van Wijk and van Hasselt (1990), Dau and Zaharieva (2009), Blankenship et al. (2011), Renger (2012a).

## Detailed structure of photosystem II and the Mn_4_CaO_5_ cluster

Each monomer of the cyanobacterial PSII core has 17 integral membrane proteins, 3 peripheral (extrinsic) proteins, and more than 80 cofactors (35 Chl, 2 Pheo, 2 PQ, 2 heme, 12 Cars, 25 lipids, 2 Cl^−^, the Mn_4_CaO_5_ cluster, non-heme Fe, bicarbonate, Y_Z_, Y_D_). The detailed protein composition and the arrangement and structure of the redox-active cofactors of PSII are shown in Fig. 5 (panels *a* and *b*, respectively) and described in its legend. Interestingly, in addition to Chls, several Cars are also present here. The Cars contribute to light harvesting and its regulation, as well as to the protection against the ROS (Frank et al. 2000; Telfer 2002; Braslavsky and Holzwarth 2012; Demmig-Adams et al. 2014; Telfer 2014; Derks et al. 2015). Remarkably, the crystal structure shows the presence of more than 1300 water molecules per PSII monomer (see dots in Fig. 5a and b); they are mostly located in the regions close to the cytoplasm and in the lumenal extensions of PSII (Umena et al. 2011; Sakashita et al. 2017b). On the lumenal side, three water-filled channels have been identified (Vassiliev et al. 2012; Hussein et al. 2021) that connect the lumen with the site of water oxidation, the Mn_4_CaO_5_ cluster (see the blue arrows in the zoomed region of Fig. 5b). These regulate the water access and facilitate the egress of protons from this site. Moreover, there are also channels at the plastoquinone site (Q_B_ site) for proton transfer from the outer water phase and for the entry of PQ and for the ‘ejection’ of PQH_2_ (Guskov et al. 2009; Ho 2012).

**Fig. 7:**
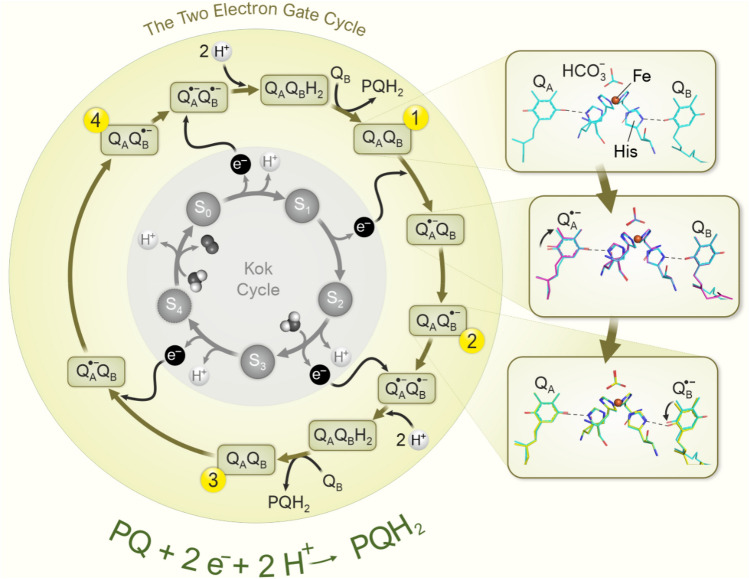
Schematic overview of the two-electron reduction of plastoquinone at the acceptor side of PSII, also known as “Two Electron Gate,” and its relationship with the Kok cycle for oxygen evolution (the inner gray circle, see Fig. 8) at the donor side of PSII. Structural changes between Q_A_Q_B_ (in the dark), Q_A_^•−^Q_B_ (50 µs after 1st light flash), and Q_A_Q_B_^•−^ (400 µs after 1st light flash) are based on room temperature time-resolved diffraction studies (Kern et al. 2018; Ibrahim et al. 2020). The numbers in yellow circles indicate the number of light flashes counting from the dark-adapted S_1_ state with fully oxidized Q_A_ and Q_B_. The structures of the Q_A_Q_B_-region were generated by employing the coordinates of the PDB codes 6W1O (for Q_A_Q_B_), 6W1R (for Q_A_^•−^Q_B_), and 6W1P (for Q_A_Q_B_^•−^). The figure is adapted from Agrisera Educational Poster 5 (Shevela et al. 2021a). Reproduced with permission of Agrisera AB (Sweden)

The *Oxygen Evolving Complex* (OEC) or *Water Oxidizing Complex* (WOC) with its Mn_4_CaO_5_ cluster is buried deeply within the PSII complex; this protects it from decomposition by too much water or cellular reductants (Hillier and Wydrzynski 2008; de Lichtenberg et al. 2021). As seen from the zoomed region in Fig. 5b, the Mn_4_CaO_5_ cluster is ligated by amino acids from the D1 protein and the inner antenna protein CP43 (Loll et al. 2005; Umena et al. 2011). The direct ligand sphere is composed of six carboxylate groups (D1-Asp170, D1-Glu189, D1-Glu333, D1-Asp342, CP43-Glu354, and the C-terminus of the D1 protein, D1-Ala344) and of one histidine residue (D1-His332). The coordination sphere of the metal ions is completed by four water molecules (W1 and W2 bound to Mn4, and W3 and W4 bound to the Ca ion; see Figs. 5b and 7b) and 5 connecting oxo-bridges (Umena et al. 2011). Remarkably, in the dark-stable state, the Mn_4_CaO_5_ cluster has one open coordination site at Mn1 (Yano et al. 2006; Siegbahn 2011; Kern et al 2018; reviewed in Pantazis 2018; Yano and Yachandra 2014; Cox et al. 2020). Another histidine (D1-His337) is H-bonded to one of the µ3-oxo-bridges of the cluster. We note that the negative charges of the oxo-bridges, the hydroxo ligand (W2) as well as of the 6 carboxylate ligands balance the positive charges of the Mn ions (Krewald et al. 2015; Siegbahn 2018; Ugur et al. 2016; Yamaguchi et al. 2022). The carboxylate ligands also provide stability by bridging generally between two metal ions. The Mn_4_CaO_5_ cluster is one of the few examples in Nature where redox active, and inactive metals are present within one cofactor (Krewald et al. 2016). The overall shape of the Mn_4_CaO_5_ cluster is difficult to describe but has been compared to a “*chair*” (Umena et al. 2011) or described as an open Mn_3_Ca cubane with one attached external (dangling) Mn.

Importantly, the WOC is more than the Mn_4_CaO_5_ cluster, as the protein and water environment are crucial for the catalytic activity. While one side of the cluster is essentially dry, the other connects to an extended H-bonding network of more than 10 water molecules that are mostly ligated by the protein. These ensure efficient proton release into the channels and controlled water access to the substrate binding sites. Two Cl^−^ ions bind in the vicinity of the Mn_4_CaO_5_ cluster (green spheres in Fig. 5b, Umena et al. 2011), of which Cl1 is crucial for proton egress via the Cl1-channel (Yocum 2008; Rivalta et al. 2011; Suzuki et al. 2013; Debus 2014; Guerra et al. 2018; Ibrahim et al. 2020; Hussein et al. 2021; Kaur et al. 2021; Imaizumi and Ifuku 2022).

Figure 5 shows the PSII structure of the thermophilic cyanobacterium *Thermosynechococcus elongatus*, obtained at room temperature by serial crystallography at an X-ray free-electron laser (XFEL) (Ibrahim et al. 2020). The arrangement of protein subunits and cofactors was initially resolved using X-ray crystallography with a resolution ranging from 3.8 Å to 1.9 Å (Zouni et al. 2001; Kamiya and Shen 2003; Ferreira et al. 2004; Loll et al. 2005; Guskov et al. 2009; Umena et al. 2011; Tanaka et al. 2017). Advances in single-particle cryo-EM have provided exciting new information as they have resolved high-resolution structures of PSII supercomplexes under various (physiological) conditions. As crystallization is not required in this method, not only PSII from cyanobacteria (Kato et al. 2021; Yu et al. 2021; Zabret et al. 2021; Gisriel et al. 2022), but also from higher plants have been explored (Wei et al. 2016; Su et al. 2017; Cao et al. 2018; Graça et al. 2021). Moreover, structures of PSII from various groups of algae have also become available (Ago et al. 2016; Burton-Smith et al. 2019; Nagao et al. 2019; Shen et al. 2019; Sheng et al. 2019).

Further Reading: Hillier and Wydrzynski (2008), Debus (2014), Ibrahim et al. (2020).

## Light harvesting and excitation energy transfer to photosystem II reaction center

The light reactions in PSII begin with the capture of *photons* by the *pigments*. Depending on the organism, the pigments include Chl *a*, other Chls, Cars, or PBs. Most of these pigments are bound to the LHCs in specific geometric arrangements, which are illustrated in Figs. 4 and 5 (Mirkovic et al. 2017; Malý and van Grondelle 2018; Green 2019; Adir et al. 2020; Croce 2020; Croce and van Amerongen 2020; Müh and Zouni 2020; Lokstein et al. 2021; Sui 2021). After the absorption of a photon, an electron of the pigment molecule is promoted from the highest occupied molecular orbital to specific excited (unoccupied) singlet states of the same molecule. Thereby, the light energy is converted into excitation energy (van Grondelle and Novoderezhkin 2006; Renger 2009; Mirkovic et al. 2017; Bennett et al. 2019; Lambrev et al. 2020). Because of the proximity of other antenna pigment molecules with the same or similar electronic energy levels, the excited singlet state energy has a high probability to be transferred to a neighboring pigment molecule. Such transfer can occur via two distinct mechanisms. If the coupling between the pigment molecules of the LHCs is strong, distribution of the excitation energy occurs in a *coherent* way. This means that the excitation energy (*exciton*) is *delocalized* among all the pigment molecules of the antenna [*Delocalized (Coherent) Exciton Model*] (Ishizaki and Fleming 2012; Fassioli et al. 2014). By contrast, if the coupling between the neighboring pigment molecules is weak, the excitation energy gained by the absorption of a photon is localized on a single pigment molecule. Here, the probability of the energy transfer strongly depends on the overlap of the emission spectrum of the donor pigment molecule and the absorption spectrum of the acceptor pigment, and, in addition, on the orientation of these neighboring pigments. In such case, the *Förster Hopping Mechanism* (usually called the *Förster Resonance Energy Transfer*) applies, in which the excitation energy is transferred by *hopping* from one pigment to the next (Jang et al. 2004; Şener et al. 2011) until the RC Chl *a* molecules are reached, where the primary photochemical reaction takes place (Fig. 6a). It appears that due to intrinsic disorder in proteins, the hopping mechanism dominates in light-harvesting complexes, but that groups of pigments exhibiting coherent excitation energy transfer also exist.

Further reading: van Grondelle and Novoderezhkin (2006), Renger (2009), Mirkovic et al. (2017).

## Charge separation and stabilization—bridging the time scales

As described above, at the end of the light-harvesting process, the excitation energy is transferred to a special ensemble of photoactive RC Chl *a* molecules (P_D1_, P_D2_, Chl_D1_, and Chl_D2_) that are symmetrically arranged in the D1 and D2 protein subunits of the PSII RC, and traditionally defined as P680 (see Figs. 3–6). However, to discuss the charge separation process in more detail, we will, in the following discussion, refer to specific pigments rather than to P680.

Since the electronic energy levels of the photoactive pigment molecules in the PSII RC are similar, the excitation energy equilibrates rapidly (within ~ 1 ps) between them and even pheophytin (Pheo_D1_). The *primary charge separation*, *i.e.*, the transfer of an electron from one molecule to another, begins from the singlet excited state of one of the RC Chls and ends with the electron being located on Pheo_D1_ and the hole on P_D1_ (Fig. 6a, i*.e.*, with the state P_D1_^•+^ Pheo_D1_^•−^). It has been proposed that this process occurs via two different pathways (Mamedov et al. 2015; Romero et al. 2017). In the P_D1_ pathway (lower path in Fig. 6a), charge separation occurs between P_D1_ and P_D2_ producing P_D2_^•+^P_D1_^•–^. By electron and hole transfer, the P_D1_^•+^ Chl_D1_^•–^ charge pair is formed thereafter, which is followed by further electron transfer to Pheo_D1_. In contrast, the Chl_D1_ pathway (upper path in Fig. 6a) starts with charge separation between Chl_D1_ and Pheo_D1_, followed by electron transfer from P_D1_ to Chl_D1_^•+^. Interestingly, both pathways may take place in the same PSII RC, and which path is taken appears to depend on the protein conformation, indicating the similarity of the energy levels of the four pigments in P680, and their modulation by the protein environment, although recent computations indicate that the Chl_D1_ pathway may be preferred (Sirohiwal et al. 2020).

This primary charge separation occurs within ~ 3 ps (Fig. 6b). Importantly, the quantum efficiency of the overall photochemistry of PSII depends then on preventing the recombination of the charges. As described above, the high efficiency is accomplished by the rapid (~ 250 ps) transfer of the electron from Pheo_D1_^•−^ to the tightly bound quinone molecule, Q_A_, and further to the more loosely bound plastoquinone molecule, Q_B_, which acts as a two-electron acceptor. Electron transfer from Q_A_^•−^ to Q_B_ occurs with a characteristic time of 200–300 µs, while the electron transfer from Q_A_^•−^ to Q_B_^•−^ is slower (700 µs) because of the negative charge of Q_B_^•−^ (de Wijn and van Gorkom 2001). This step is likely coupled to a proton uptake so that Q_B_H^•−^ is formed, which is subsequently protonated to Q_B_H_2_ (for reviews on a role of bicarbonate in this step, see Govindjee and Van Rensen 1993; Shevela et al. 2012; Müh and Zouni 2013). After Q_B_H_2_ (which is PQH_2_) leaves the side, the empty Q_B_ site is filled by an oxidized PQ molecule from the PQ pool (see Figs. 1 and 6b). This takes up to 20 ms and thereby is the slowest reaction event among all light-induced reactions of the photosynthetic electron transfer chain.

The high oxidizing potential of the cation radical P_D1_^•+^ (midpoint potential of ~  + 1.25 V) allows P_D1_^•+^ to sequentially withdraw electrons from the Mn_4_CaO_5_ cluster via the tyrosine residue (Y_Z_) of the D1 protein (see Figs. 3, 5b, 6b). The rate of electron transfer from Y_Z_ to P680^•+^ is multiphasic with halftimes in the 20 ns to 35 μs range. This is likely due to a sequence of relaxation processes involving proton transfer to the nearby D1-His190 residue as well as large-scale proton relaxation via the hydrogen bonding network around the Mn_4_CaO_5_ cluster. Thus, the relative amplitude of the μs phase varies with S state (Renger 2012a). Y_Z_^•^, with a redox potential of about 1.1 V (Rappaport et al. 2002; Ishikita and Knapp 2006), oxidizes the Mn_4_CaO_5_ cluster with half times ranging from 0.1 to 2 ms (Renger 2012a; Styring et al. 2012). As illustrated in Fig. 6b, PSII utilizes about 50% of the energy corresponding to a 680 nm photon for ensuring fast kinetics and a high quantum efficiency for the production of PQH_2_ and O_2_, and to thereby minimize harmful recombination reactions.

 Further reading: Rappaport et al. (2002), Renger (2012a), Romero et al. (2017).

## Chemical reactions

### Plastoquinone reduction: the two-electron gate

PSII is linked to the Cyt *b*_6_*f* complex via a pool of lipophilic hydrogen atom carriers, the PQ molecules, which are mobile within the thylakoid membrane. These PQ molecules play an important role in oxygenic photosynthesis by connecting the electron transport with the proton transfer reactions across the photosynthetic membrane. In PSII, one plastoquinone molecule is permanently bound at the Q_A_ site. Due to the special ligand sphere at this binding site, Q_A_ can accept only one electron and no proton. A second PQ molecule can bind transiently at the Q_B_ site, where it can accept, sequentially, two electrons via Q_A_/Q_A_^•–^ and two protons from the stroma/cytoplasm. As described, the resulting PQH_2_ is released into the photosynthetic membrane. Since two electrons generated in subsequent photochemical reactions are needed for the formation and release of one PQH_2_ molecule, the reduction of PQ at the Q_B_ site is often referred to as *The Two Electron Gate* (Bouges-Bocquet 1973; Velthuys and Amesz 1974; Müh et al. 2012). The sequence of reactions taking place in the two-electron gate is depicted in detail in Fig. 7.

The structural details of the initial steps of this cycle have been resolved by recording x-ray crystal structures of PSII at various times after a flash given to dark-adapted PSII crystals (Kern et al. 2018; Ibrahim et al. 2020). Figure 7 (right side) shows the structures of Q_A_, Q_B_, and the non-heme iron for the dark-state and two snapshots obtained at 50 µs and 400 µs after the first flash. The non-heme iron is ligated by four histidine residues, of which two are provided by the D1 and two by the D2 protein. The ligand sphere is completed by the binding of a bicarbonate (HCO_3_^−^) ion (Umena et al. 2011), which facilitates the formation of Q_B_^−^ and its subsequent protonation (reviewed in McConnell et al. 2012; Shevela et al. 2012). Reduction of Q_A_ leads to a small rotation of its head group, as seen in the 50 µs structure. By 400 µs, the electron is completely transferred to Q_B_, as can be seen by a reversal of the Q_A_ rotation, and a rotation of Q_B_^•–^ similar to that seen upon Q_A_^•–^ formation. Q_B_^•–^ is not protonated, but instead the negative charge appears to be compensated by protonation of a nearby amino acid side chain.

Under conditions in which Q_A_ is reduced and cannot be oxidized, for example, when the PQ pool in the membrane is fully reduced (under high light stress), bicarbonate is expelled from its binding site at the non-heme iron (Brinkert et al. 2016; Shevela et al. 2020). This changes the redox potentials of the PSII cofactors so that the charge recombination pathways are changed in a way that the formation of long-lived triplet radicals is minimized, which reduces the formation of ROS (Brinkert et al. 2016). This protection mechanism is specific for PSII of oxygenic photosynthesis. The evolutionary-related bacterial RC has instead of bicarbonate a glutamate-ligand to the non-heme iron, and thus does not have this regulatory mechanism (Michel and Deisenhofer 1988; Shopes et al. 1989; Wang et al. 1992).

Numerous herbicides, used in agriculture, are known to cause inhibition of photosynthesis due to their binding at the Q_B_ site of PSII. Blockage of the PQ reduction by bound herbicide results in the lack of electron delivery into PSI and the carbon fixation cycle. This increases recombination processes, which generate ROS that cause serious damage to PSII and thereby may contribute to the death of the plant (Oettmeier 2003).

Further reading: McConnel et al. (2012), Müh et al. (2012), Shevela et al. (2012).

### Water oxidation—the four electron gate

#### Period-four oscillation and the Kok model

**Fig. 8:**
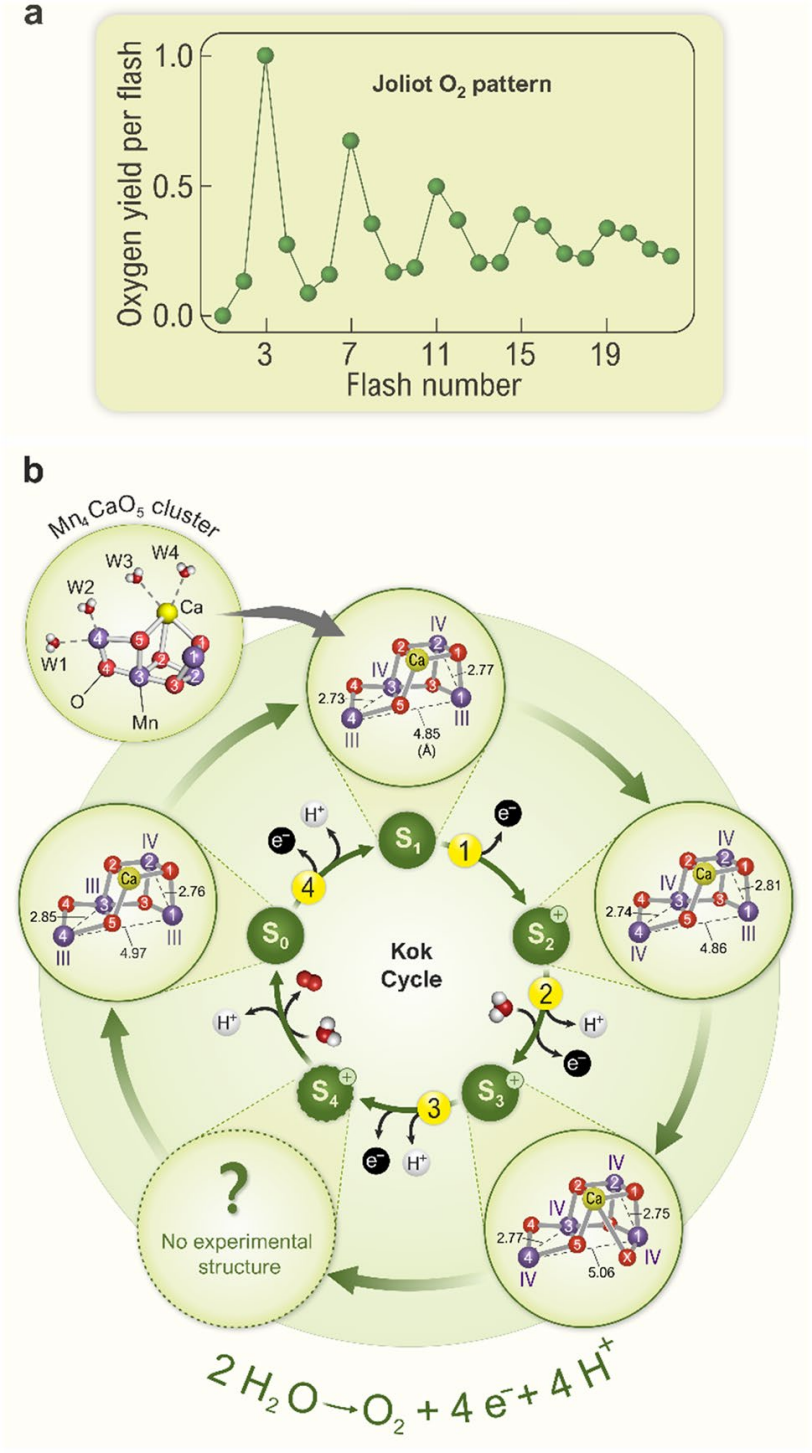
Flash-induced oxygen evolution pattern (FIOP) from dark-adapted PSII thylakoids (**a**), the Kok cycle that explains the damped period-four oscillation (inner circle of panel **b**) and experimental structures of the Mn_4_CaO_5_ cluster obtained for the various S states (outer circle of panel **b**). FIOPs (data of one of the authors, JM) were originally obtained by Joliot and coworkers in 1969 (Joliot et al. 1969; Joliot 2003) and are thus also known as the* “Joliot O*_*2*_* pattern.”* Note that the first maximum of O_2_ evolution is observed after the 3rd flash, while it thereafter takes four flashes to the next maxima. It can also be seen that the period-four oscillation is less pronounced at higher flash numbers. **b** Inner circle: The Kok cycle (also known as *“Oxygen Cycle,”* *“Kok’s Clock,”* or *“The S State Cycle”*) illustrates the stepwise process of photosynthetic water oxidation and O_2_ production by the Mn_4_CaO_5_ cluster of PSII. Green arrows depict S state transitions and the numbers in yellow circles correspond to the number of light flashes from panel (**a)**. This kinetic model was originally developed by Kok and coworkers based on Joliot’s O_2_ pattern and additional experiments (Kok et al. 1970). The Kok cycle consists of five redox intermediates, the S_*i*_-states (*i* = 0, …4), where *i* is the number of oxidizing equivalent(s) stored within the Mn_4_CaO_5/6_ cluster. Upon accumulation of four oxidizing equivalents, the highly reactive S_4_ state converts into the S_0_ state, while the oxygen atoms of two (deprotonated and ligated) H_2_O molecules are oxidized and combine to form O_2_. Thus, four light-induced removal steps of electrons are needed to release four protons and one O_2_ molecule from two H_2_O molecules per each turn of the Kok clock. Note that (*i*) in the dark, the Mn_4_CaO_5/6_ cluster is mostly in the S_1_ state, which explains why the first O_2_ maximum occurs after the 3rd flash, (*ii*) the S_4_ ⟶ S_0_ transition does not require light, (*iii*) electron and proton removal steps alternate (Klauss et al. 2012), and (*iv*) there are two water binding events, namely in the S_2_ → S_3_ and the S_4_ → S_0_ transitions (Suzuki et al. 2008; Siegbahn 2009; Shen 2015; Kern et al. 2018; Kim and Debus 2019). Since in the S_1_ → S_2_ transition, no proton is released, a positive charge is accumulated at the Mn_4_CaO_5_ cluster (indicated by “ + ” sign) and, in the S_2_ and S_3_ states, a proton needs to be released first before the Mn_4_CaO_5_ cluster can be oxidized (Dau et al. 2012). The O_2_ pattern, as first shown by Joliot, is damped due to misses and double hits connected with each S state transition. Outer circle: Recent progress has allowed researchers to obtain high-resolution structures of all the *S states*, and even of some transient intermediates, but not yet of the S_4_ state (Kern et al. 2018; Suga et al. 2019; Ibrahim et al. 2020). Mn oxidation states are indicated by roman numbers. Selected atomic distances within the Mn_4_CaO_5_ cluster are given in Å. The structure of the Mn_4_CaO_5/6_ cluster with metal bound waters (W1–W4) in the S_1_ state was generated by using coordinates of the PDB code 6W1O. The figure is adapted from Agrisera Educational Poster 5 (Shevela et al. 2021a). Reproduced with permission of Agrisera AB (Sweden)

When dark-adapted PSII samples are illuminated with one flash, no O_2_ is produced and even after the second flash, only a very small amount is formed. Finally, after the third flash, a large O_2_ signal is observed, while upon further flashes, the O_2_ yields diminish again until upon the 7th flash the next maximum occurs (Fig. 8a). This period-four oscillation continues with maxima after the 11th and 15th flashes, but it damps out over time so that finally all flashes produce the same amount of O_2_. This phenomenon was discovered by Pierre Joliot and coworkers in 1969 (Joliot et al. 1969). It is the iconic evidence that PSII has a storage unit, as we now know the Mn_4_CaO_5_ cluster, which first stores four oxidizing equivalents, produced in four successive flash-induced charge separations between P680 and Pheo_D1_, before O_2_ is formed from two deprotonated, Mn-bound oxygen atoms derived from water molecules. While the period-four oscillation was described by Pierre Joliot, it was Bessel Kok and his coworkers who, after performing important additional experiments, devised the *S*_*i*_* state model* that describes the accumulation of oxidizing equivalents (central circle in Fig. 8b). This model is usually referred to as the *Kok model* or the *Kok clock* (Kok et al. 1970). Here, *S* stands for *state*, and the index *i* signifies the number of stored oxidizing equivalents, which varies from 0 to 4. In this model, each flash advances the Mn_4_CaO_5_ cluster into the next higher S_*i*_ state until it spontaneously returns back from S_4_ to S_0_ due to O_2_ formation and release. We note that the S_4_ state is a postulated transient intermediate state and has not yet been experimentally observed.

The fact that the first maximum of O_2_ production is observed already after the 3rd flash indicates that the singly oxidized state, S_1_, is the dark-stable state, while all other states are transformed, in the dark, by redox reactions with other cofactors within PSII into this state. The S_2_ and S_3_ states are reduced, in the seconds to minutes time scales, to S_1_ by charge recombination with electrons from the electron acceptor side (Q_B_^−^, Q_B_H_2_) or by electron donation from tyrosine Y_D_ (see Figs. 3 and 5b). The S_0_ state, on the other hand, is oxidized to S_1_, which occurs in the 10’s of min time scale by electron transfer to the oxidized form of Y_D_, which is an unusually stable neutral radical, abbreviated as Y_D_^ox^ or Y_D_^•^ (Styring and Rutherford 1988; Vass and Styring 1991; Messinger et al. 1993; Messinger and Renger 1994, 2008; Isgandarova et al. 2003).

In contrast to the lower S_*i*_ states, the S_4_ state is a highly reactive state. In this state, the four oxidizing equivalents required to extract four electrons from two water molecules are ready to be used, and, thus, O_2_ formation occurs without further energy input within 1–2 ms. O_2_ formation has been found to be an exergonic reaction, meaning that it is irreversible even if an O_2_ pressure of several bars is applied (Haumann et al. 2008; Kolling et al. 2009; Shevela et al. 2011).

Kok and coworkers additionally introduced a *double hit* parameter to explain the small O_2_ yield observed already after the second flash, and a *miss* parameter for explaining the dampening of the *flash-induced oxygen evolution pattern* (FIOP). The double hit parameter depends on the flash profile and thus reflects the probability that centers advance twice in one flash, *i.e.,* by two S_*i*_ states. The miss parameter, on the other hand, gives the percentage of centers that do not advance in a flash. Typically, the miss parameter is of the order of 10%, thus indicating a quantum efficiency of the reaction in PSII of up to 90%. For simplicity, the miss parameter is often assumed to be constant for all S state transitions. However, the miss parameter is a consequence of redox equilibria between all the cofactors of PSII, and is thus expected to be both flash number and S_*i*_ state dependent (Shinkarev and Wraight 1993; de Wijn and van Gorkom 2002; Han et al. 2012; Suzuki et al. 2012; Pham and Messinger 2016; Han et al. 2022).

The Kok model has passed the test of time, and period-four oscillations have also been observed with many other techniques probing the reactions of the WOC. As will be described below, over the past 50 years it has been possible to move from the kinetic scheme describing FIOPs to a molecular level understanding of the reactions at the Mn_4_CaO_5_ cluster (outer ring of Fig. 8b).

Further reading: Kok et al. (1970), Messinger and Renger (2008); for a historical discussion of the kinetic models, see Mar and Govindjee (1972).

#### Channels

When looking at the structure of PSII, one obvious feature is that the site of water oxidation is buried deep inside the PSII complex. Thus, for allowing water access and proton egress, specific channels are needed (reviewed in Ho 2012). Three channel networks have been identified and variously named (Murray and Barber 2007; Ho and Styring 2008; Gabdulkhakov et al. 2009; Guskov et al. 2009; Vassiliev et al. 2010; Umena et al. 2011; Sakashita et al. 2017a). The Cl1 channel has been shown to be crucial for proton egress (Hussein et al. 2021; Dau et al. 2022), but also the O4 channel may have that function during the S_0_ ⟶ S_1_ transition (Sakashita et al. 2017a; Hussein et al 2021). All the three channels (Fig. 5b) are suggested to contribute to various extent in supporting water access at rates that do not limit water oxidation (Vassiliev et al. 2012). However, recent experimental work supports specifically the O1 channel and Ca to be involved in water access and binding in the S_2_ ⟶ S_3_ transition (Noguchi and Sugiura 2002; Suzuki et al. 2008; Kern et al. 2018; Suga et al. 2019; Ibrahim et al. 2020; Hussein et al. 2021), while most present computational studies prefer water access via the O4 or Cl1 channels and thereby the Mn4 site as water entry point into the Mn_4_CaO_5_ cluster. Importantly, all channels severely reduce water access (Vassiliev et al. 2012; de Lichtenberg et al. 2021), which is crucial for the stability of the Mn_4_CaO_5_ cluster since Mn^III^ ions can undergo rapid ligand exchange, a problem well known from molecular water oxidation catalysts (Gil-Sepulcre and Llobet 2022). Indeed, removing one or more of the extrinsic proteins of PSII leads to destabilization of the Mn_4_CaO_5_ complex, as do elevated temperatures. It thus seems that the existing channels are optimized to guarantee stability of the Mn_4_CaO_5_ cluster against protein ligand exchange with water, while providing water access and proton egress fast enough that PSII performance is not limited (Wydrzynski et al. 1996; de Lichtenberg et al. 2021).

 Further reading: Vassiliev et al. (2012), Ho (2012), de Lichtenberg et al. (2021).

#### Proton release pattern and proton coupled electron transfer

If after each flash the Mn_4_CaO_5_ cluster would loose one electron but no proton, the positive charge of the cluster would increase upon each S_*i*_ state advancement. This is indeed observed for the S_1_ ⟶ S_2_ transition: while the S_1_ state is neutral, the S_2_ state has a positive charge and is, thus, denoted by a plus sign (S_2_^+^ state). Removing the next electron against a positive charge requires a much higher oxidation potential, which poses a problem because in each flash the same redox potential is generated, about 1.1 V at the level of Y_Z_^•^/Y_Z_. Thus, before the S_2_^+^ state can be oxidized to the S_3_^+^ state, one proton must be released from the cluster (see Kok model in Fig. 8b) (Krishtalik 1986; Hoganson and Babcock 1997; Siegbahn 2009; Klauss et al. 2012; Allgöwer et al. 2022). This principle is known as *Proton Coupled Electron Transfer* (PCET) and is now recognized to be highly important for efficient catalysis in biology and chemistry (Cukier 2002; Hammes-Schiffer 2006; Huynh and Meyer 2007; Weinberg et al. 2012; Koper 2013; Tyburski et al. 2021). As expected, also in the S_3_^+^Y_Z_^•^ state, a proton needs to be expelled, before the S_4_ state and subsequently the S_0_ state, O_2_, and H^+^ are formed. The S_0_ state is neutral, and the available data suggest that during the S_0_ ⟶ S_1_ transition the electron is removed first, which is then followed by proton release (Klauss et al. 2015). Thus, proton release has, at near neutral pH, a pattern of approximately 1: 0: 1: 2 for the S_0_ ⟶ S_1_⟶ S_2_ ⟶ S_3_ ⟶ S_0_ transitions. This proton release pattern is pH dependent due to electrostatic effects on the residues around the Mn_4_CaO_5_ cluster (Lavergne and Junge 1993; Schlodder and Witt 1999; Suzuki et al. 2009). Easily exchangeable (mobile) bicarbonate ions are suggested to enhance functionality of the Mn_4_CaO_5_ cluster by shuttling protons produced during water oxidation into the lumen (Shutova et al. 2008; Ulas and Brudvig 2010; Shevela et al. 2013; Koroidov et al. 2014).

Further reading: Klauss et al. (2012), Tyburski et al. (2021), Allgöwer et al. (2022).

#### Substrate water binding

Two substrate water molecules need to bind during each round of the Kok cycle. In many older schemes, both these molecules are indicated to bind during the S_4_ ⟶ S_0_ transition, *i.e.,* concomitant with O_2_ release. Substrate water exchange experiments, using H_2_^18^O-labeling and mass spectrometric detection of O_2_, have indeed established that both these molecules are present in the OEC already in the S_2_ state in different chemical environments (Messinger et al. 1995; Hillier and Wydrzynski 2001; Cox and Messinger 2013; Nilsson et al. 2014a). By combining the substrate water exchange kinetics with structural information gained from X-ray crystallography, EXAFS and EPR experiments, as well as from Density Functional Theory (DFT) calculations, it has been demonstrated that the central O5-bridge of the Mn_4_CaO_5_ cluster (Fig. 8b) is one of the two substrates (slowly exchanging substrate water, W_s_) (Messinger 2004; Kulik et al. 2007; Rapatskiy et al. 2012; Cox and Messinger 2013; Pérez Navarro et al. 2013; Siegbahn 2013). The identity of the second, fast exchanging substrate water (W_f_) has not yet been uniquely established, since the exchange rate in the S_2_ state is limited by water diffusion barriers in the channels connecting the Mn_4_CaO_5_ cluster with the bulk water. Thus, both W2 and W3, or a protein ligated water in the OEC, remain as candidates in the S_2_ state. However, in the S_3_ state, the exchange of W_f_ slows down and thus it must be bound more tightly. Binding of W_f_ to Mn in the S_3_Y_Z_^•^ state (and thus also in the S_3_ state) was proven by the finding that its exchange is arrested in the S_3_Y_Z_^•^ state (Fig. 8b) (Nilsson et al. 2014b; de Lichtenberg and Messinger 2020; de Lichtenberg et al. 2021). Experiments on thermophilic cyanobacteria have established that one water molecule binds during the S_2_ ⟶ S_3_ transition and forms a new oxo-bridge in the S_3_ state, marked as X in Fig. 8b (Cox et al. 2014; Suga et al. 2017, 2019; Kern et al. 2018; Ibrahim et al. 2020). This additional oxo or hydroxo bridge between Ca and Mn1 is known as Ox or O6 in the literature. One possible scenario is, therefore, that W_f_ is bound to Ca (as W3) in the S_2_ state, while it forms the new Ox/O6 bridge in the S_3_ state (de Lichtenberg et al. 2021). Alternatively, it has been suggested that W_f_ binds as W2 to Mn4 in the S_2_ state, and rotates, during the S_2_ ⟶ S_3_ transition, into the O5 position, while the original O5 becomes the Ox/O6 of the S_3_ state (pivot/carousel mechanisms; Retegan et al. 2016; Wang et al. 2017). Importantly, due to water binding in the S_2_ ⟶ S_3_ transition, only one water needs to be replenished during the S_4_ ⟶ S_0_ transition.

 Further reading: Cox and Messinger (2013), Pantazis (2018), Lubitz et al. (2019), Ibrahim et al. (2020).

#### Mn oxidation states

After a long debate, it is now well established that in the S_1_ state the Mn oxidation states are Mn_4_^III,III,IV,IV^, and it is largely agreed that all S state transitions up to the S_3_ state involve Mn^III^ → Mn^IV^ oxidation state changes (outer circle in Fig. 8b) (Messinger et al. 2001; Haumann et al. 2005b; Kulik et al. 2007; Dau and Haumann 2008; Siegbahn 2009; Yano and Yachandra 2014; Krewald et al. 2015; Cheah et al. 2020). In contrast, no conclusive experimental data are yet available for the S_4_ state. Nevertheless, the S_3_ ⟶ S_4_ transition most likely leads to an oxyl radical formation, *i.e.,* to the beginning of water oxidation. This is supported by DFT calculations (Siegbahn 2009; Li and Siegbahn 2015; Allgöwer et al. 2022), and by the absence of experimental evidence for Mn^V^ formation (Haumann et al. 2005a). However, Yamaguchi and coworkers suggest that the ligation of O6 to the Ca ion suppresses oxyl formation at the expense of an increased Mn^V^ character on the Mn1 ion, leading to O-O bond formation via the Ca-assisted concerted bond switching (CBS) mechanism (Yamaguchi et al. 2019 and 2022; Shoji et al. 2019) . Nevertheless, also O–O bond formation or oxyl radical formation at the level of the S_3_ state, in a fraction of centers within a dynamic equilibrium, is still being discussed (Renger 2012a; Isobe et al. 2016, 2019; Pushkar et al. 2018; Corry and O’Malley 2021). Further research is needed to clarify these points.

 Further reading: Haumann et al. (2005a), Krewald et al. (2015), Cheah et al. (2020), Yamaguchi et al. (2022).

#### Structural changes of the WOC

The outer circle in Fig. 8b shows the structure(s) of the Mn_4_CaO_5_ cluster in the S_0_ → S_3_ states as determined by serial crystallography at physiological temperatures (Kern et al. 2018). The Mn ions are shown in purple, while the oxygen bridges and Ca are displayed as red and yellow spheres, respectively. Characteristic changes of the Mn–Mn and Mn–Ca distances are given in Å. The S_0_ → S_1_ transition leads to a shortening of the Mn3-Mn4 and Mn1–Mn4 distances by about 0.1 Å, which is consistent with deprotonation of one of the two oxo-bridges connecting Mn3 and Mn4, likely of the O5. This would imply that O5 is a hydroxide bridge in the S_0_ state, while it is an oxo-bridge in the S_1_ state, which would be consistent with the oxidation of Mn3 in this step and thus to a drop in pKa of this group (Robblee et al. 2002; Siegbahn 2009). In contrast, the oxidation of Mn4 in the S_1_ → S_2_ transition is not connected to metal distant changes, likely because all bridges are already deprotonated (no proton is released to the bulk in that transition; however, if W2 is a water molecule (Yamaguchi et al. 2022) and not a hydroxide (Pantazis 2018), an internal proton relocation remains a possibility).

The S_2_ → S_3_ transition is one of the most complex steps in the reaction cycle. After Y_Z_^•^ formation, a proton needs to be released before the cluster can be oxidized. This deprotonation step involves the W1 water (Siegbahn 2009). Next, Mn1 shall be oxidized to Mn^IV^. However, Mn1 is the only 5-coordinate Mn ion of the Mn_4_CaO_5_ cluster, and the Mn^IV^ ions strongly ‘prefer’ to be 6-coordinated. Thus, a water molecule is suggested to associate with the Mn_4_CaO_5_ cluster concomitant with Mn1 oxidation, which also involves an internal proton transfer from this water molecule to the previously deprotonated W1 ligand on Mn4 (Kern et al. 2018; Ibrahim et al. 2020; Allgöwer et al. 2022). Thus, in the S_3_ state, we have a Mn_4_CaO_6_ cluster (Suga et al. 2017; Kern et al. 2018). This brings Ox (or O6) and O5 close to each other, possibly setting up the geometry required for O–O bond formation (Siegbahn 2009). To allow the Ox insertion, the Mn_4_CaO_5/6_ cluster needs to undergo an expansion by which, specifically, the Mn1–M4 distance increases by 0.2 Å. In contrast, the Mn1–Mn2 distance shrinks by 0.06 Å, reflecting oxidation of Mn1(Kern et al. 2018; Ibrahim et al. 2020). Although at present three different pathways are being discussed as to how this water insertion really takes place (Capone et al. 2016; Retegan et al. 2016; Ugur et al. 2016; Vinyard et al. 2016; Kim and Debus 2017; de Lichtenberg et al. 2021; Allgöwer et al. 2022), a direct insertion of water via the Ca ion appears most probable due to water motions observed in the O1 channel that indicate water delivery to the Ca site (Kern et al. 2018; Suga et al. 2019; Ibrahim et al. 2020; Hussein et al. 2021).

In addition to the structural changes of the Mn_4_CaO_5/6_ cluster during S state transition discussed above, evidence for at least two conformations for each S state has been obtained from EPR experiments under various conditions. The most prominent example is the low-spin and high-spin EPR signals of the S_2_ state, which have been assigned to the open cubane structure (shown in Fig. 8b) and to a second conformation, respectively. Additional experiments and calculations are needed for obtaining the precise structures and catalytic relevance of these alternative conformations (Retegan et al. 2016; Isobe et al. 2016; Boussac et al. 2018; Corry and O’Malley 2019; Pushkar et al. 2019; de Lichtenberg and Messinger 2020; Drosou et al. 2021; Chrysina et al. 2019; Drosou and Pantazis 2021).

 Further reading: Kern et al. (2018), Pantazis (2018), Lubitz et al. (2019).

#### O–O bond formation and O_2_ release

**Fig. 9:**
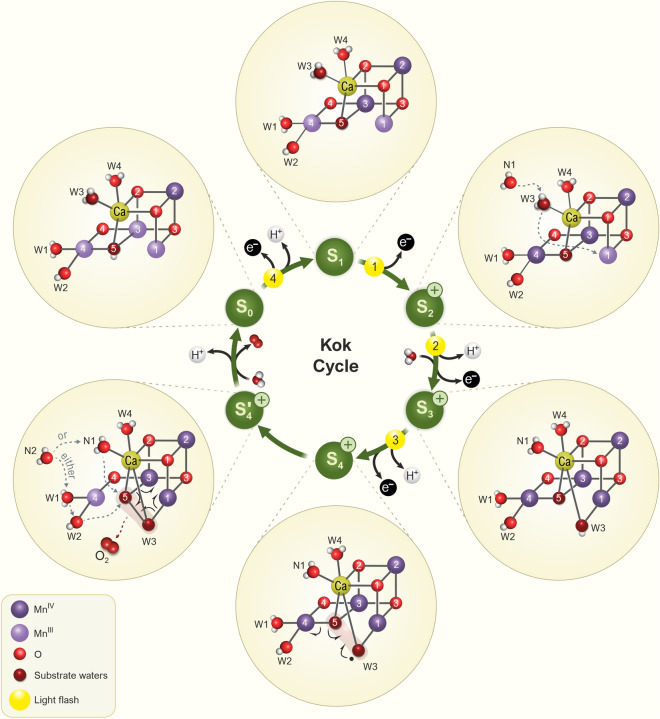
Illustration of a possible mechanism of photosynthetic water oxidation and dioxygen formation in PSII. The outer circles represent schematically the dominant structures of the Mn_4_CaO_5/6_ cluster from cyanobacterial PSII in the S_0_ − S_3_ states as determined by X-ray diffraction (see Fig. 8), while the structures in the S_4_ and S_4_’ states are based on structural models suggested by computational studies for the O − O formation (Siegbahn 2009; Li and Siegbahn 2015). Thus, for the S state transitions up to S_3_ all is the same as in Fig. 8, only that here the two ‘substrate waters’ (term used independently of protonation state) are tentatively assigned to O5 (there is very good evidence for it; see Messinger 2004; Cox and Messinger 2013; Rapatzkiy et al. 2012) and W3 (an alternative would be W2 or a water within the OEC) in the S_1_ and S_2_ states. For the S_2_ → S_3_ transition, it is assumed that W3 binds to the open coordination site of Mn1, while that is oxidized, and that the original W3 binding site at Ca is refilled by a new water molecule (N1). At this stage, all Mn ions are in the Mn^IV^ oxidation state, and both substrates are bonded to Mn. The next light-induced transition, S_3_ ⟶ S_4_, involves the oxidation of the substrate water (marked with black dot on W3, now in the ‘Ox’ position; an alternative would be Mn_1_^V^ formation). In the S_4_ state, re-arrangement of the electrons of the chemical bonds (shown by black half arrows) leads to a rapid conversion into a new conformation, which has a complexed peroxide (–O5–W3–). This new state is depicted as S_4_’ state (or “S_2_^P^,” where P stands for “peroxide”). The S_4_’ ⟶ S_0_ transition involves the formation (black half arrows) and release of O_2_ (red dashed arrow), the binding of one new water molecule (N2) to one of the indicated sites (either at W1 or at N1 as indicated by gray dashed arrows), and removal of one proton (Siegbahn 2009; Li and Siegbahn 2015). Here, a pre-bound water ligand (W2 or N1) is proposed to occupy the empty O5-binding site and to release one proton in the process (Messinger 2004; de Lichtenberg et al. 2021). As a result, the O5-bridge is protonated in the S_0_ state (Kulik et al. 2007; Lohmiller et al. 2017). The S_0_ ⟶ S_1_ transition involves the oxidation of Mn3 and release of a proton, resulting in a deprotonated O5-bridge. According to several biophysical studies, all the S states appear to exist in equilibrium with other conformations, which may be crucial intermediates for S state transitions and substrate water exchange, as well as possible alternatives for O–O bond formation (Boussac et al. 2018; Pantazis 2018; Yamaguchi et al. 2019; de Lichtenberg and Messinger 2020; de Lichtenberg et al. 2021; Guo et al. 2021). The numbers displayed in the yellow circles on the arrows depict the number of flashes given to the dark-adapted PSII. Mn^III^ and Mn^IV^ ions are colored individually. The two substrate ‘water molecules’ are colored as dark red spheres, while all other oxygen atoms are colored in red. Hydrogen atoms are shown as white spheres

The oxidation of S_3_ to S_4_ by Y_Z_^•^ is again a PCET step due to the positive charge of the S_3_ state (S_3_^+^ in Figs. 8b and 9). Experimentally, a lag phase was observed between the Y_Z_^•^ formation and Mn reduction, and the O_2_ release, which has been assigned to a proton release—likely again W1 is deprotonated (Rappaport et al. 1994; Razeghifard and Pace 1999; Haumann et al. 2005a; Siegbahn 2009; Gerencsér and Dau 2010). In contrast, thus far, no clear experimental evidence for the existence of S_4_ proper (oxidation of the cluster, *e.g.,* of Ox as shown in Fig. 9) has been obtained, possibly due to its short lifetime. Thus, the sequence of reactions leading to O–O bond formation relies presently on the DFT calculations and chemical reasoning.

A possible mechanism for O–O bond formation is shown in Fig. 9. It identifies the two substrate waters as O5 and W3 (marked in dark red) by assuming the arguably simplest of the water insertion steps during the S_2_ → S_3_ transition, which is the direct binding of W3 to the open coordination site at Mn1, coupled to the replenishing of the original W3 binding site at Ca^2+^ by a new water molecule (N1 in Fig. 9) (Ugur et al. 2016; Kern et al. 2018; Kim and Debus 2019; Ibrahim et al. 2020; de Lichtenberg et al. 2021; Allgöwer et al. 2022).

For the O–O bond formation, after the proton removal step, discussed above, an oxyl radical is formed at the Ox site, which is coupled to an internal proton transfer to the deprotonated W1 (Siegbahn 2006, 2009; Shoji et al. 2018a; Allgöwer et al. 2022). To form the O–O bond, two electrons with opposite spins are required, which, due to the spin coupling in the Mn_4_CaO_6_ cluster of the S_3_ state, can be provided by the radical on W3 (in Ox position) and by one of the electrons of the bond between O5 and Mn4, since the spins on Mn4 are favorably aligned to accept the other electron from this bond (see half arrows in the S_4_ state of Fig. 9) (Siegbahn 2006). The resulting complexed peroxide intermediate is displayed as S_4_’ state in Fig. 9. From here, the double bond between the two oxygen atoms is formed under reduction of Mn1 and Mn3, so that Mn1, Mn3, and Mn4 are in the oxidation state Mn^III^ in the S_0_ state, while Mn2 remains in the oxidation state Mn^IV^ throughout the reaction cycle. As O_2_ leaves the cluster, the central O5-bridge needs to be replenished. This may be best done by a pre-bound water ligand (Messinger 2004; Cox and Messinger 2013; Li and Siegbahn 2015; Shoji et al. 2018b). In Fig. 9, we show two possible pathways for this process. Like in the S_2_ → S_3_ transition, the O5-binding site may be filled by W3 bound to Ca, or by W2 bound to Mn, and the second new water molecule, N2, is suggested to replenish the respective terminal ligand site.

Interestingly, the slow kinetic of the S_3_ → S_4_ → S_0_ transition appears to be connected to large entropic contributions rather than a high activation barrier. This indicates that a large number of conformational states may need to be sampled to find the optimal configuration of the Mn_4_CaO_5_ cluster and the H-bonding network to allow generation of the S_4_ state as well as O_2_ formation and release (Zaharieva and Dau 2019; Dau et al. 2022). Lastly, the S_3_ → S_4_ → S_0_ transition is irreversible due to its large driving force (Haumann et al. 2008; Kolling et al. 2009; Shevela et al. 2011; Nilsson et al. 2016).

We emphasize that the precise mechanism of water oxidation is still under investigation and that several options are still being discussed: see, for example, O–O bond formation in the closed cubane conformation (Messinger 2004; Nilsson et al. 2014a; Li and Siegbahn 2015), concerted bond switching instead of radical coupling (Shoji et al. 2019; Yamaguchi et al. 2022), geminal coupling at Mn4 (Kusunoki 2007; Zhang and Sun 2018), nucleophilic attack of W3 on W2 (Sproviero et al. 2008; Vinyard et al. 2015; see however Siegbahn 2017), or O–O bond formation in the S_3_ state (Renger 2012a; Isobe et al. 2016, 2019; Pushkar et al. 2018; Corry and O’Malley 2021).

Further Reading: McEvoy and Brudvig (2006), Siegbahn (2009), Dau et al. (2010), Renger (2012a), Pantazis (2018), Cox et al. (2020), de Lichtenberg et al (2021), Allgöwer et al. (2022), Yamaguchi et al (2022); for review of the historic development, see Junge (2019).

#### Principles of biological water oxidation

The WOC in PSII is unique; it is unmatched by synthetic complexes in the ability to split water using a catalyst made of earth-abundant elements. This is true for both its stability and the required low potential of + 1.1 V (Y_Z_^•^/Y_Z_) to drive all the necessary transitions. Some key points mentioned above are summarized as follows:

(1) Water access is limited by the protein matrix for the stabilization of the Mn_4_CaO_5_ cluster, and for minimizing harmful side reactions.

(2) The ligands of the Mn_4_CaO_5_ cluster are negatively charged (except water ligands and one histidine ligand) and mostly bridge two metal centers. This allows for stabilizing the Mn^III^ and Mn^IV^ oxidation states, which is important for the stability and catalytic efficiency of the WOC.

(3) A H-bonding network is in place that allows efficient redox leveling *via* PCET.

(4) The Mn_4_CaO_5/6_ cluster allows storage of three oxidizing equivalents on Mn, while the fourth oxidation creates a terminal oxygen radical (or Mn^V^-oxo), which immediately initiates O−O bond formation in a pre-set and optimized geometry.

(5) The Mn_4_CaO_6_ cluster provides an optimal spin coupling for low energy barrier O−O bond formation.

(6) Ca serves as water binding hub that supports the H-bonding network and rapid water insertion into open binding sites at Mn during the S_2_ ⟶ S_3_ and possibly the S_4_ ⟶ S_0_ transitions. Ca may also tune the acidity of its water ligands to the optimal value for this insertion and for templating O−O bond formation.

(7) The Mn_4_CaO_5/6_ cluster remains flexible, with at least two possible conformations in each oxidation state, which supports water insertion, oxidation state changes as well as O_2_ formation and release.

(8) O_2_ formation and release are highly entropic, allowing for the irreversibility of the reaction at a low overpotential (activation barrier).

In spite of the complexity, we are optimistic that the improved design of synthetic water oxidation catalysts, according to these principles, will allow us to obtain highly active, stable, and scalable water oxidation catalysts suitable for sustainable energy applications.

## Data Availability

This review does not contain any original data.

## Abbreviations

ADP: Adenosine diphosphate
APC: Allophycocyanin
ATP: Adenosine triphosphate
Car: Carotenoid
Chl: Chlorophyll
cryo-EM: Cryo electron microscopy
Cyt b_6_f: Cytochrome *b*_6_*f* complex
DFT: Density functional theory
EET: Excitation energy transfer
Fd: Ferredoxin
FIOP: Flash-induced oxygen evolution pattern
FNR: Ferredoxin-NADP^+^ reductase
LHCII: Light-harvesting complex II
Mn_4_CaO_5_: Manganese-calcium-oxygen complex
NADP^+^/NADPH: Nicotinamide-adenine dinucleotide phosphate (oxidized/reduced forms)
OEC: Oxygen-evolving complex
PBS: Phycobilisomes
PB: Phycobilin
Pc: Plastocyanin
PE/PC: Phycoerythrin/phycocyanin
Pheo: Pheophytin
PQ/PQH_2_: Mobile plastoquinone molecules (oxidized/reduced forms)
PSI: Photosystem I
PSII: Photosystem II
P680: Primary electron donor of photosystem II that includes Chl *a* molecules
ROS: Reactive oxygen species
Q_A_/Q_B_: Primary/secondary plastoquinone electron acceptors
RC: Reaction center
WOC: Water-oxidizing complex
Y_D_/Y_Z_: Redox-active tyrosine D/Z
